# The membrane protein ANKH is crucial for bone mechanical performance by mediating cellular export of citrate and ATP

**DOI:** 10.1371/journal.pgen.1008884

**Published:** 2020-07-08

**Authors:** Flora Szeri, Stefan Lundkvist, Sylvia Donnelly, Udo F. H. Engelke, Kyu Rhee, Charlene J. Williams, John P. Sundberg, Ron A. Wevers, Ryan E. Tomlinson, Robert S. Jansen, Koen van de Wetering

**Affiliations:** 1 Department of Dermatology and Cutaneous Biology, Jefferson Institute of Molecular Medicine and PXE International Center of Excellence in Research and Clinical Care, Sidney Kimmel Medical College, Thomas Jefferson University, Philadelphia, Pennsylvania, United States of America; 2 Translational Metabolic Laboratory, Department Laboratory Medicine, Radboud University Medical Center, Nijmegen, The Netherlands; 3 Division of Infectious Diseases, Department of Medicine, Weill Cornell Medicine, New York, New York, United States of America; 4 Cooper Medical School of Rowan University, Camden, New Jersey, United States of America; 5 The Jackson Laboratory, Bar Harbor, Maine, United States of America; 6 Department of Orthopedic Surgery, Thomas Jefferson University, Philadelphia, Pennsylvania, United States of America; Universitatsklinikum Regensburg, GERMANY

## Abstract

The membrane protein ANKH was known to prevent pathological mineralization of joints and was thought to export pyrophosphate (PPi) from cells. This did not explain, however, the presence of ANKH in tissues, such as brain, blood vessels and muscle. We now report that in cultured cells ANKH exports ATP, rather than PPi, and, unexpectedly, also citrate as a prominent metabolite. The extracellular ATP is rapidly converted into PPi, explaining the role of ANKH in preventing ankylosis. Mice lacking functional Ank (*Ank*^*ank/ank*^ mice) had plasma citrate concentrations that were 65% lower than those detected in wild type control animals. Consequently, citrate excretion via the urine was substantially reduced in *Ank*^*ank*/*ank*^ mice. Citrate was even undetectable in the urine of a human patient lacking functional ANKH. The hydroxyapatite of *Ank*^*ank*/*ank*^ mice contained dramatically reduced levels of both, citrate and PPi and displayed diminished strength. Our results show that ANKH is a critical contributor to extracellular citrate and PPi homeostasis and profoundly affects bone matrix composition and, consequently, bone quality.

## Author summary

Biallelic inactivating mutations in the progressive ankylosis gene (*ANKH/Ank*, humans/mice) cause a severe form of arthritis, primarily affecting joints of hands, feet and the spine. Low extracellular concentrations of the mineralization inhibitor pyrophosphate underlie the joint calcification in mice and humans that are devoid of functional Ank or ANKH, respectively. It was previously thought that ANKH mediates the release of pyrophosphate from the cytosol to the extracellular environment. We here show, however, that PPi is formed in the extracellular environment by Ectonucleotide Pyrophosphatase Phosphodiesterase 1 (ENPP1), from ATP released via ANKH. Untargeted metabolomics further revealed that ANKH provides a pathway for the release of nucleoside triphosphates in general and, unexpectedly, the Krebs-cycle intermediate citrate. Mice lacking Ank activity (*Ank*^*ank/ank*^ mice), have substantially reduced concentrations of citrate in their plasma and urine. Citrate was even undetectable in urine of a human patient lacking functional ANKH. Bones of *Ank*^*ank/ank*^ mice have reduced mechanical strength and their bone matrix contains dramatically reduced levels of PPi and citrate. Our data demonstrate that ANKH/Ank crucially contributes to the disposition of citrate and PPi *in vivo* and profoundly affects bone quality. Extracellular citrate is ubiquitous, and additional phenotypes are therefore expected to be associated with absence of Ank activity.

## Introduction

Physiological mineralization is essential for normal development of vertebrates, but must be restricted to specific sites of the body. Vertebrates have evolved mechanisms to allow regulated mineralization in for instance bones and teeth, but prevent mineralization of soft connective tissues [1,2]. The molecular details of the mechanism in vertebrates that restrict mineralization to specific sites of the body are incompletely characterized, however.

The *ANKH*/*Ank* (human/mouse) gene encodes a multi-span transmembrane protein involved in the prevention of pathological mineralization of cartilage and synovial fluid [3,4]. Ank, has a wide tissue distribution, with high levels of expression found in osteoblasts, prostate, skeletal muscle, brain and the cardiovascular system [1,5–7]. A naturally occurring mouse mutant, progressive ankylosis (*Ank*^*ank*/*ank*^), presents early in life with progressive ankylosis of the spine and other joints, restricting mobility and critically limiting lifespan [1]. Biallelic loss-of-function mutations in the human orthologue of *Ank*, *Ank homolog* (*ANKH)*, result in progressive small joint soft-tissue calcification, hearing loss, progressive spondyloarthropathy and mental retardation [5], clinical manifestations very similar to those observed in *Ank*^*ank/ank*^ mice[1]. In 2000, Ho *et al*. showed that medium of *Ank*^*ank*/*ank*^ fibroblasts contained reduced concentrations of the physiological mineralization inhibitor inorganic pyrophosphate (PPi), leading to the now prevailing view that ANKH transports PPi from the cytosol to the extracellular environment [1,6]. An important source of extracellular PPi is ATP, which is extracellularly converted into AMP and PPi by membrane-bound ecto-nucleotide pyrophosphatase/phosphodiesterase 1 (ENPP1) [7]. We have previously shown that ATP release mediated by the hepatic membrane protein ATP-Binding Cassette subfamily C member 6 (ABCC6) is responsible for 60–70% of all PPi present in plasma [8,9].

Here we tested if release of ATP also underlies most of the PPi found in the extracellular milieu of ANKH-containing cells. Moreover, given its wide tissue distribution, we hypothesized ANKH has functions beyond regulation of extracellular PPi homeostasis, and applied global metabolite profiling [10] on medium of HEK293-ANKH cells to gain a comprehensive overview of metabolites extruded by cells in an ANKH-dependent manner. Our results provide new and unexpected insights into the substrate spectrum and anti-mineralization properties of ANKH and also show that ANKH has functions beyond inhibition of pathological mineralization as it, for instance, determines bone quality by regulating bone matrix composition.

## Results

### HEK293-*ANKH* cells release ATP into the extracellular environment

**Fig 1:**
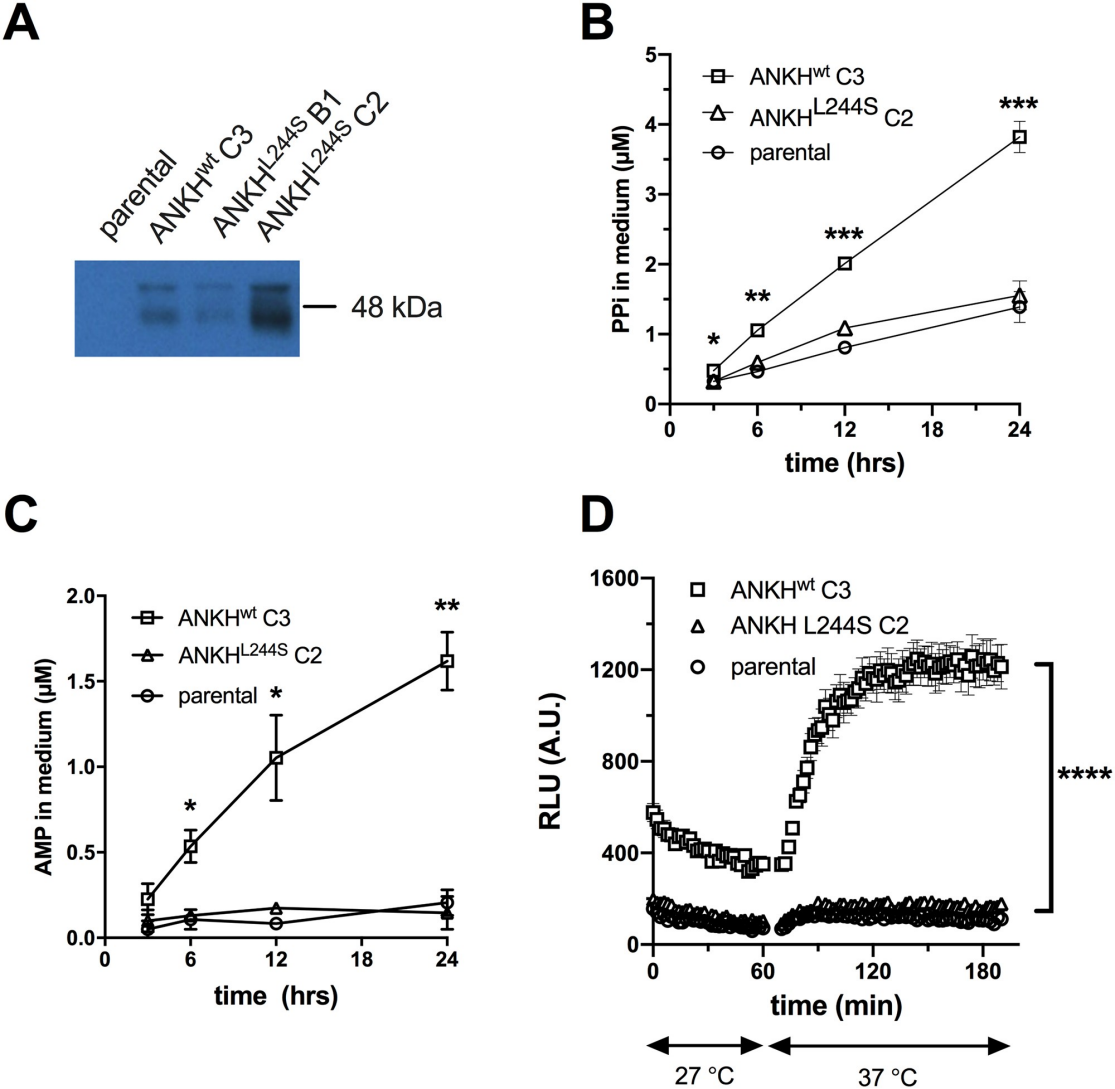
HEK293-*ANKH*^*wt*^ cells release ATP, which is rapidly converted into pyrophosphate (PPi) and AMP. Detection of ANKH in HEK293 parental, HEK293-*ANKH*^*wt*^ and HEK293-*ANKH*^*L244S*^ cells by immunoblot analysis (**A**). Concentrations of pyrophosphate (PPi) (**B**) and AMP (**C**) were quantified enzymatically and followed in medium samples of HEK293 parental, HEK293-ANKH^wt^ and HEK293-*ANKH*^*L244S*^ cells over the course of 24 hours. ATP release by HEK293 parental, HEK293-*ANKH*^*wt*^ and HEK293-*ANKH*^*L244S*^ cells was followed in real time using a luciferase-based assay (**D**). Results of representative experiments performed in triplicate are shown. In panels B and C data are expressed as mean +/- SD. Panel D shows mean +/- SEM. Statistical significance for ANKH^wt^ C3 vs parental and ANKH^wt^ C3 vs ANKH^L244S^ C2, was calculated by 2-way ANOVA, with Bonferroni’s correction, using GraphPad Prism version 8.4.2. * p < 0.05, ** p < 0.01, *** p < 0.001, **** p < 0.0001.

To study the function of ANKH *in vitro*, we first generated several HEK293 cell lines overproducing wild type ANKH (ANKH^wt^) and ANKH^L244S^, a pathogenic loss-of-function mutant which still routes normally to the plasma membrane [5]. As shown in Fig 1A, endogenous ANKH was not detectable in parental HEK293 cells by immunoblot analysis, whereas high levels of ANKH protein were found in cells overexpressing *ANKH*^*wt*^. The loss-of-function *ANKH*^*L244S*^ mutant was also abundantly expressed, and clone C2 which produced levels of the mutant protein higher than those detected in the HEK293-*ANKH*^*wt*^ cells was used for further analysis (Fig 1A). First, we measured PPi levels in the medium of these cells over a 24-h time period and showed that PPi accumulated at higher levels in medium of HEK293-*ANKH*^*w*t^ cells than in medium of HEK293-*ANKH*^*L244S*^ or control HEK293 cells (Fig 1B), confirming earlier reports that demonstrated the involvement of ANKH in extracellular PPi homeostasis [1]. Medium of an independent HEK293-ANKH^*L244S*^ clone (B1) (see Fig 1A) did not contain PPi concentrations higher than those found in the HEK293 parental cells (S3A Fig), confirming that *ANKH*^*L244S*^ is a clear loss of function mutant. We have previously shown that ENPP1 produced by HEK293 cells converts extracellular ATP into AMP and PPi [8]. Consequently, to determine what part of the PPi found in medium of *ANKH*^*wt*^ cells might be derived from extracellular ATP, converted by ENPP1 into AMP and PPi, AMP concentrations were quantified in the culture medium. As shown in Fig 1C, a clear time-dependent increase in AMP concentrations was detected in medium of HEK293-*ANKH*^*wt*^ cells, while medium of non-transfected HEK293 parental cells or cells producing the loss-of-function *ANKH*^*L244S*^ mutant contained only very little AMP. PPi and AMP concentrations in medium of *ANKH*^*wt*^ cells were within the same range (1–2 μM after 12 hours, compare panels B and C of Fig 1) and the ratio of PPi to AMP was very similar to that previously reported for HEK293 cells overproducing ABCC6, a plasma membrane protein involved in the release of ATP [8]. We attribute the somewhat lower abundance of AMP than PPi to further metabolism of AMP and the generation of PPi from other nucleoside triphosphates (NTPs) also released into the culture medium via ANKH (see below). A luciferase-based real-time ATP efflux assay was also carried out and confirmed that ANKH is involved in cellular ATP release (Fig 1D). Only HEK293-*ANKH*^*wt*^ cells showed robust ATP efflux, whereas release from HEK293-*ANKH*^*L244S*^ cells was indistinguishable from non-transfected parental HEK293 cells in these assays. Collectively, these data indicate that HEK293-*ANKH*^*wt*^ cells release ATP, which is subsequently extracellularly converted into AMP and PPi.

### Culture medium of HEK293-*ANKH*^*wt*^ cells contains large amounts of nucleoside monophosphates (NMPs)

**Fig 2:**
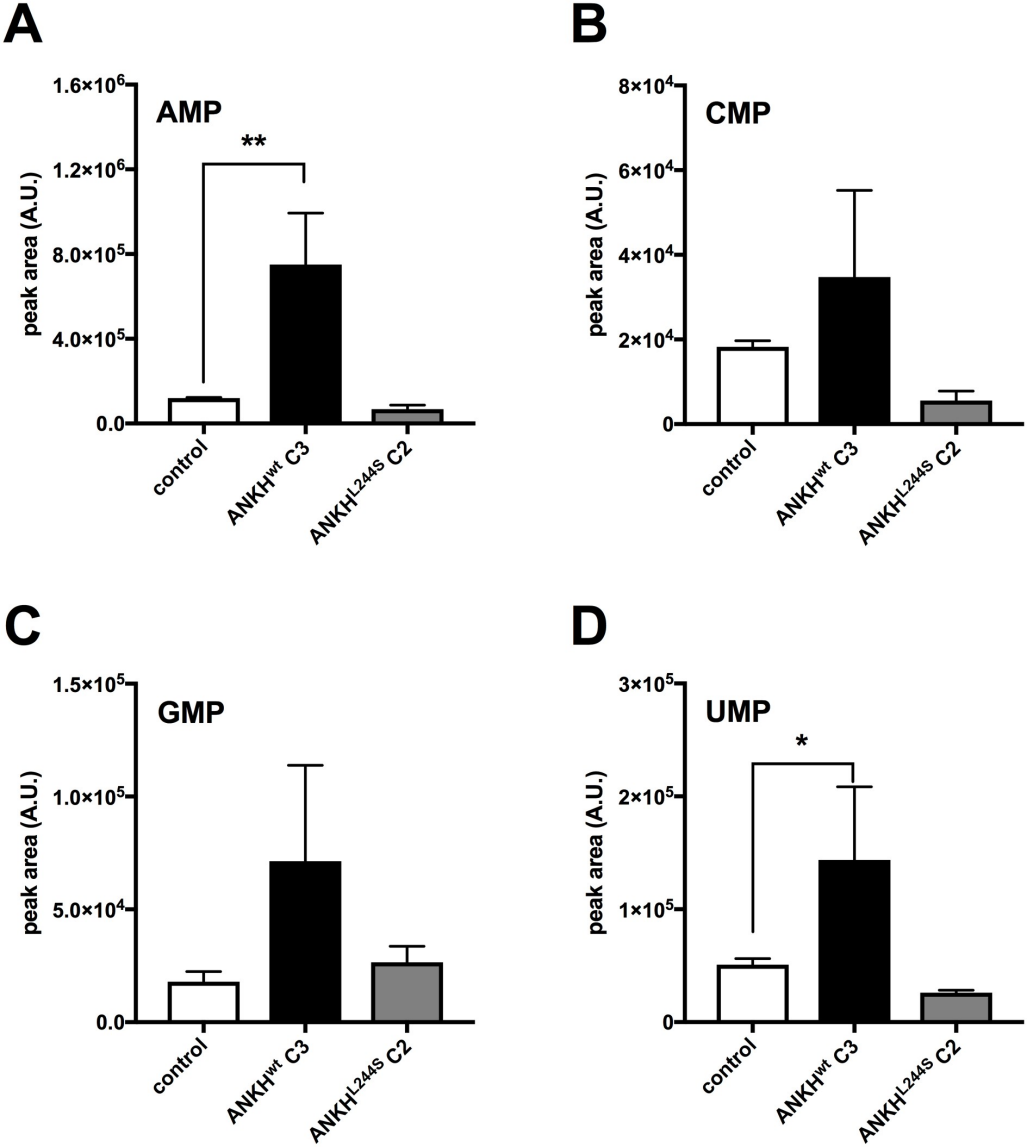
Medium of HEK293-*ANKH*^*wt*^ cells contains large amounts of nucleoside monophosphates. LC/MS-based global metabolite profiling was applied to 24-hour medium samples of HEK293 parental, HEK293-*ANKH*^*wt*^ and HEK293-*ANKH*^*L244S*^ cells. The relative abundance of masses corresponding to AMP (**A**), CMP (**B**), GMP (**C**) and UMP (**D**) were determined. Authentic standards were used to confirm the identity of NMPs. Data are expressed as mean +/- SD of an experiment performed in triplicate. Statistical significance was determined by one-way ANOVA with * p < 0.05, ** p < 0.01.

In addition to ATP, ENPP1 can convert various other nucleoside triphosphates (NTPs) into their respective nucleoside monophosphate (NMP) and PPi. Our previous work has shown that ENPP1 activity in HEK293 cells is high [8]. We therefore used liquid chromatography/mass spectrometry (LC/MS)-based global metabolite profiling to determine if ANKH also provides a pathway for release of other NTPs. Substantially elevated levels of AMP, CMP, GMP and UMP were detected in the culture medium of HEK293-*ANKH*^*wt*^ cells compared to non-transfected parental and HEK293-*ANKH*^*L244S*^ cells (Fig 2A–2D), For AMP and UMP differences between non-transfected and HEK293-*ANKH*^*wt*^ cells reached statistical significance. These results support the hypothesis that ANKH provides a previously unanticipated pathway for cellular NTP release. Based on the levels of PPi, AMP and other NMPs detected in the culture medium, we estimate that cellular NTP release underlies at least 70% of the ANKH-dependent accumulation of PPi in the culture medium (for calculation see materials and methods section) of the PPi detected in medium of the HEK293-ANKH^wt^ cells.

### HEK293-ANKH cells release the TCA cycle intermediates citrate, succinate, and malate into the culture medium

**Fig 3:**
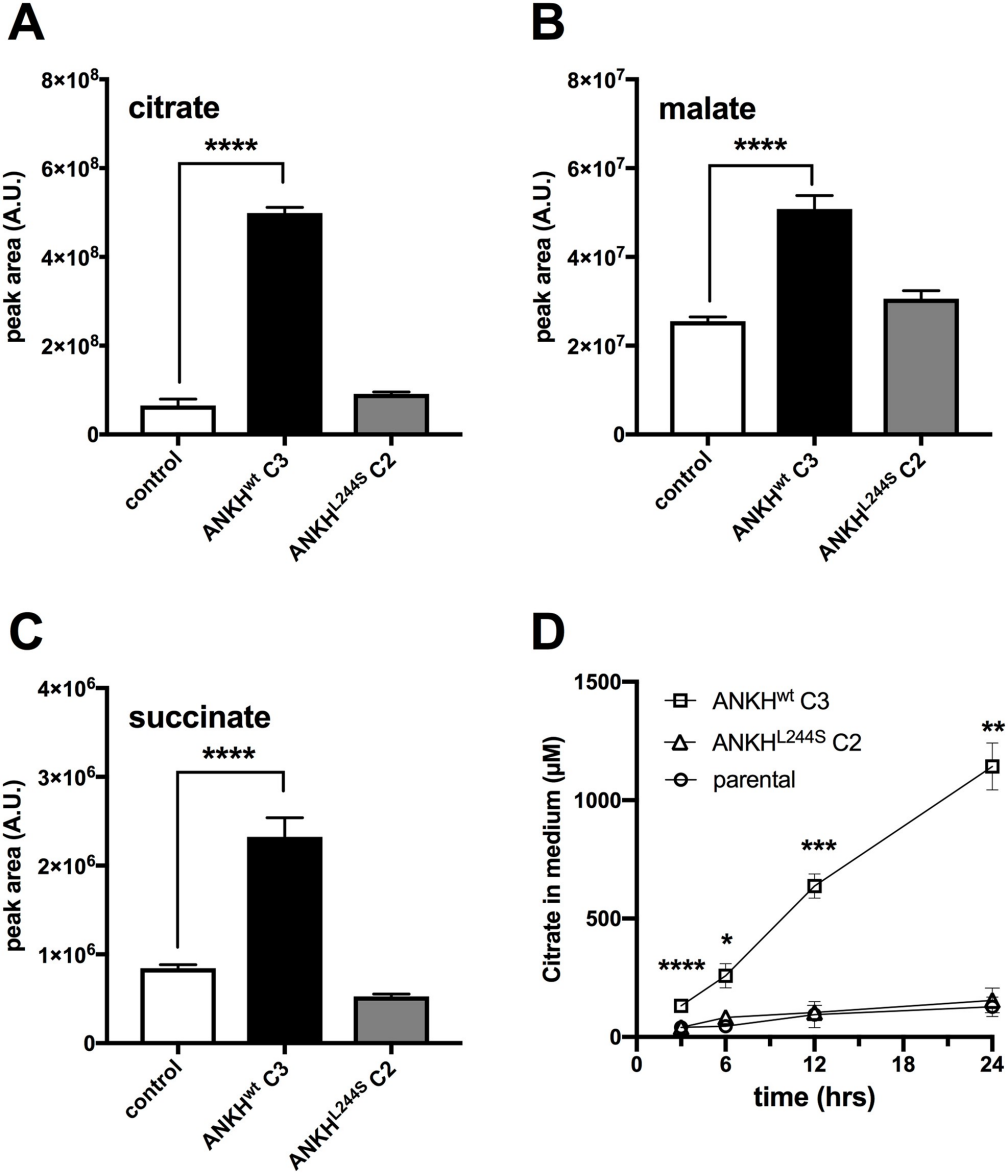
Medium of HEK293-*ANKH*^*wt*^ cells contains large amounts of citrate, succinate and malate. LC/MS-based global metabolite profiling was applied to 24-hour medium samples of HEK293 parental, HEK293-*ANKH*^*wt*^ and HEK293-*ANKH*^*L244S*^ cells. The relative abundance of masses corresponding to citrate (**A**), malate (**B**) and succinate (**C**) were determined. Authentic standards were used to confirm the identity of the Krebs-cycle intermediates. Using an enzymatic assay, citrate concentrations were followed for 24 hours in (**D**). Data are expressed as mean +/- SD of an experiment performed in triplicate. Statistical significance was determined by one-way ANOVA (panels A, B and C) or two-way-ANOVA (panel D). * p < 0.05, ** p < 0.01, *** p < 0.001, **** p < 0.0001.

The global metabolite profiling experiments also revealed that the calcium chelator citrate specifically accumulated in the culture medium of HEK293-*ANKH*^*wt*^ cells (Fig 3A). Because global metabolite profiling experiments only provide relative metabolite levels, we also quantified citrate levels by LC/MS in 24-hour medium samples and found that approximately 1 mM citrate (2.5 μmol/24 hrs) was present in medium of HEK293-*ANKH*^*wt*^ cells, while it was almost undetectable in medium of HEK293 control and HEK293-*ANK*^*L244S*^ cells. To put this in perspective, the same medium samples of HEK293-*ANKH*^*wt*^ cells contained about 4 μM PPi (Fig 1B), equivalent to the release of approximately 10 nmoles of NTPs. Thus, the amount of citrate released by the HEK293-*ANKH*^*wt*^ cells was at least 2 orders of magnitude higher than the amount of NTPs. Other metabolites found to be selectively elevated in medium of HEK293-*ANKH*^*wt*^ cells were malate (Fig 3B) and succinate (Fig 3C), although absolute levels as well as relative increase compared to control cells were clearly less than those found for citrate. To the best of our knowledge, this is the first report linking a specific membrane protein to cellular release of malate. Using an independent enzymatic assay, citrate levels in culture medium were also followed over time and as shown in Fig 3D, these experiments confirmed that citrate was present at approximately 1.1 mM in the 24-hour culture medium samples of the *ANKH*^*wt*^ cells, comparable to the concentration determined by LC/MS. Whereas the LC/MS-based assay cannot distinguish between citrate and isocitrate, the enzymatic assay specifically detects citrate. Collectively these data show that ANKH is involved in the cellular release of large amounts of citrate.

### Ank affects PPi incorporation into the bone matrix

**Fig 4:**
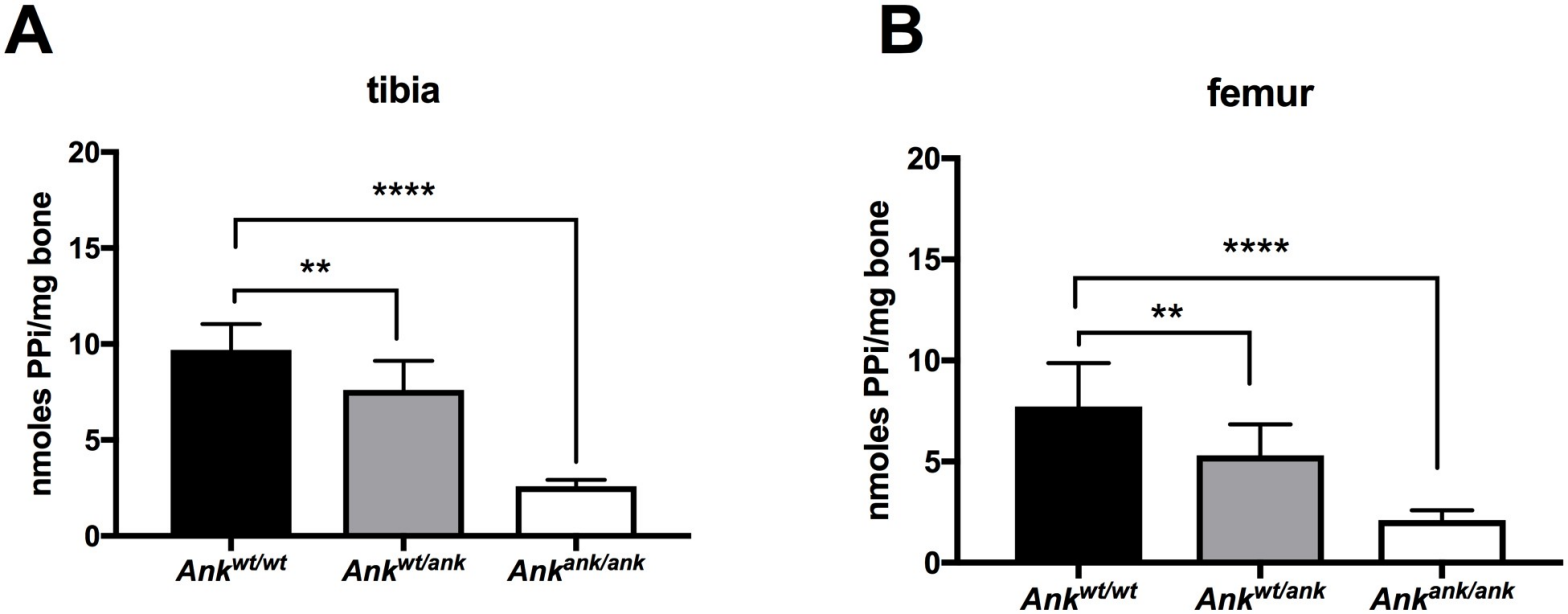
PPi content of bone tissue depends on ANK activity. Pyrophosphate content of tibiae (**A**) and femora (**B**) of wild type (n = 10), *Ank*^*wt/ank*^ (n = 10) and *Ank*^*ank*/*ank*^ (n = 8) mice. Data are expressed as mean +/- SD. Statistical significance was determined by one-way ANOVA with ** p < 0.01, **** p < 0.0001.

About 70% of the PPi found in plasma depends on ABCC6 activity [9], indicating that the contribution of ANKH to plasma PPi homeostasis is relatively minor. Consequently, instead of contributing to central PPi homeostasis in plasma, we hypothesized that Ank is important in local PPi homeostasis. Osteoblasts express *Ank* at relatively high levels [11] and the hydroxyapatite of bone contains substantial amounts of PPi [12]. To determine if Ank has a role in incorporation of PPi in bone matrix, we quantified PPi in tibiae and femora of wild type, *Ank*^*ank*/*ank*^, and mice heterozygous for *ank*. As shown in Fig 4 PPi constituted about 0.1% (weight/weight) of bone tissue in wild type mice, whereas in *Ank*^*ank*/*ank*^ mice the amount of PPi associated with bone was reduced by approximately 75%. Moreover, in mice heterozygous for *ank*, PPi levels were also moderately (by approximately 25%), but significantly reduced. These data show that Ank is a crucial factor in for the incorporation of PPi in the mineralized bone matrix.

### ANKH affects citrate disposition *in vivo*

**Fig 5:**
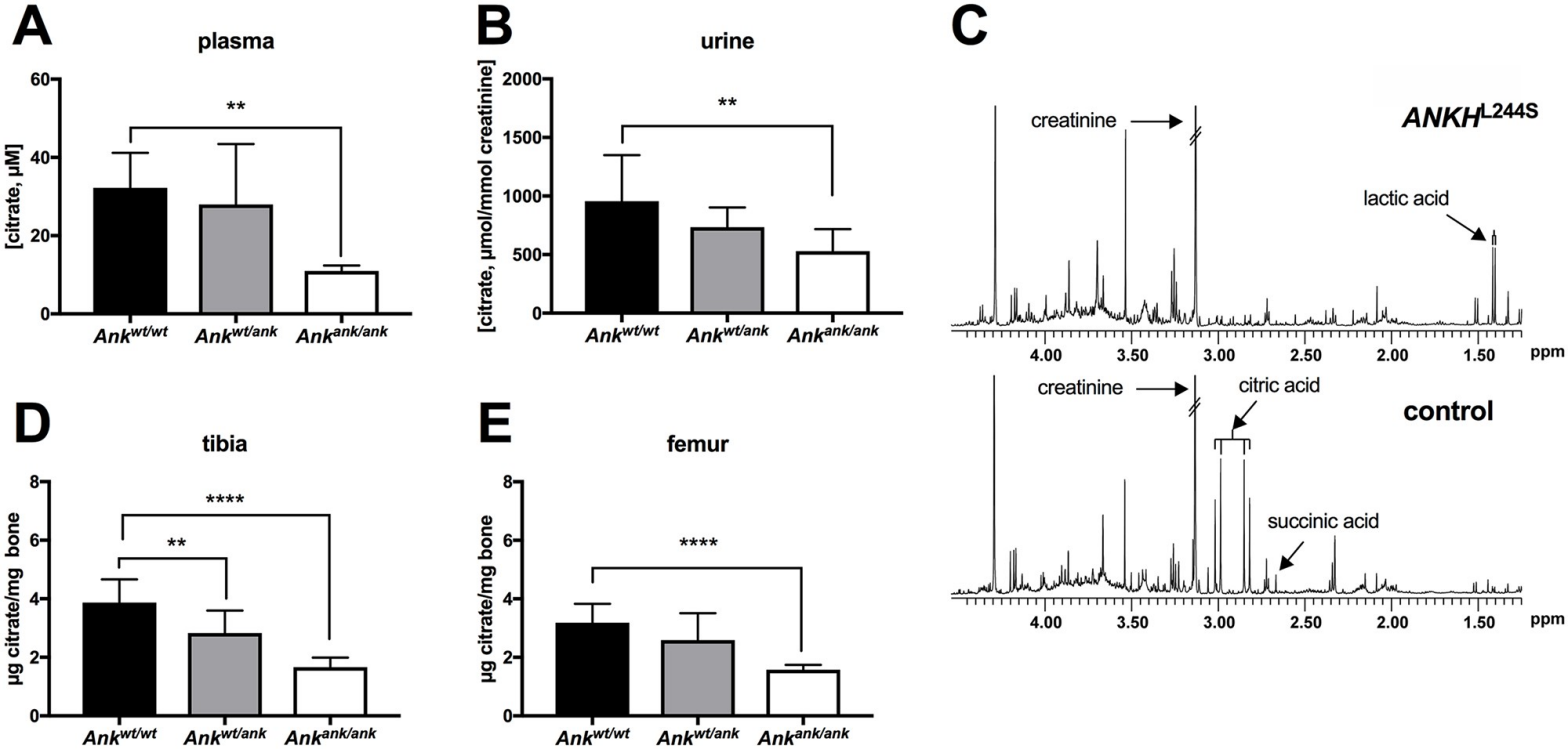
Extracellular citrate depends on ANK/ANKH activity. (**A**) Citrate plasma concentrations in wild type (*Ank*^*wt/wt*^, n = 8), heterozygous (*Ank*^*wt/ank*^, n = 8) and *Ank*^*ank*/*ank*^ (n = 8) mice. Citrate concentrations in urine of wild type (*Ank*^*wt/wt*^, n = 6), heterozygous (*Ank*^*wt/ank*^, n = 10) and *Ank*^*ank*/*ank*^ (n = 9) mice. (**C)** Urine of a patient with biallelic inactivating mutations in *ANKH* is virtually devoid of citrate. NMR spectra of urine of a patient with biallelic pathogenic *ANKH*^L244S^ mutations (**C**, upper panel). A representative sex- and age-matched control urine sample contained 370 μmol/mmol creatinine (**C**, lower panel). Spectra are scaled on creatinine. Citrate resonates as a typical AB-system (2.98 ppm; four peaks between 2.80 and 3.05 ppm). Reference values for urinary citrate for this age group are 208–468 μmol/mmol creatinine (n = 20 healthy controls) (36). Succinate resonates as a singlet resonance at 2.66 ppm. For unknown reasons, urinary lactate was somewhat increased in urine of the patient with biallelic pathogenic *ANKHL244S* mutations (120 μmol/mmol creatinine; reference <75 μmol/mmol creatinine). Citrate content of tibiae (**D**) and femora (**E**) of wild type (*Ank*^*wt/wt*^, n = 10), heterozygous (*Ank*^*wt/ank*^, n = 10) and *Ank*^*ank*/*ank*^ (n = 10) mice. Data are expressed as mean +/- SD. Statistical significance was determined by one-way ANOVA with * p < 0.05, ** p < 0.01, *** p< 0.001, **** p < 0.0001.

Plasma contains substantial amounts of citrate [13]. We therefore determined the effect of a complete inactivation of Ank in mice on plasma citrate concentrations and as shown in Fig 5A, found that approximately 75% of citrate in plasma depended on Ank. Because citrate is also one of the most abundant organic anions in urine [14], we measure citrate excretion in *Ank*^*ank*/*ank*^ mice. As shown in Fig 5B, the *ank* mutant mice excreted approximately 40% less citrate via their urine than their wild type litter mates. The availability of an NMR spectrum of urine of a 19-year-old female patient carrying a biallelic homozygous inactivating mutation in *ANKH* (*ANKH*^*L244S*^), previously described by Morava *et al*. [5] made it possible to carry out an analysis of citrate levels. Citric acid was not detected in urine of this patient (Fig 5C, upper panel). The lower panel of Fig 5C shows the typical citrate resonance in urine of a representative age-matched control, which contained 370 μmol citrate/mmol creatinine. It is interesting to note that the succinate resonance is visible in the NMR spectrum of control urine, while its concentration is clearly much lower in urine of the patient carrying biallelic mutations in *ANKH* (Fig 5E). These data suggest that ANKH impacts the *in vivo* disposition of succinate and especially citrate in both, humans and mice. It is important to note that NMR does not allow detection of malate in urine specimen.

Citrate is one of the major organic compounds present in bone and strongly associates with hydroxyapatite [15]. With 90% of the body’s citrate content present in bone, this tissue is thought to play a central role in extracellular citrate homeostasis [16]. Therefore, we determined if bone citrate levels depend on Ank. These experiments revealed that femora and tibiae of *Ank*^*ank*/*ank*^ mice contained approximately 50% less citrate than the same bones of wild type mice (Fig 5D and 5E). Moreover, bones of mice heterozygous for *ank* also contained less citrate, which in the case of tibia was significantly lower than in wild type mice (Fig 5D). Together these data attest to the major impact of Ank on citrate homeostasis in bone.

### Material properties of bone tissue of *Ank*^ank/ank^ mice are altered

**Fig 6:**
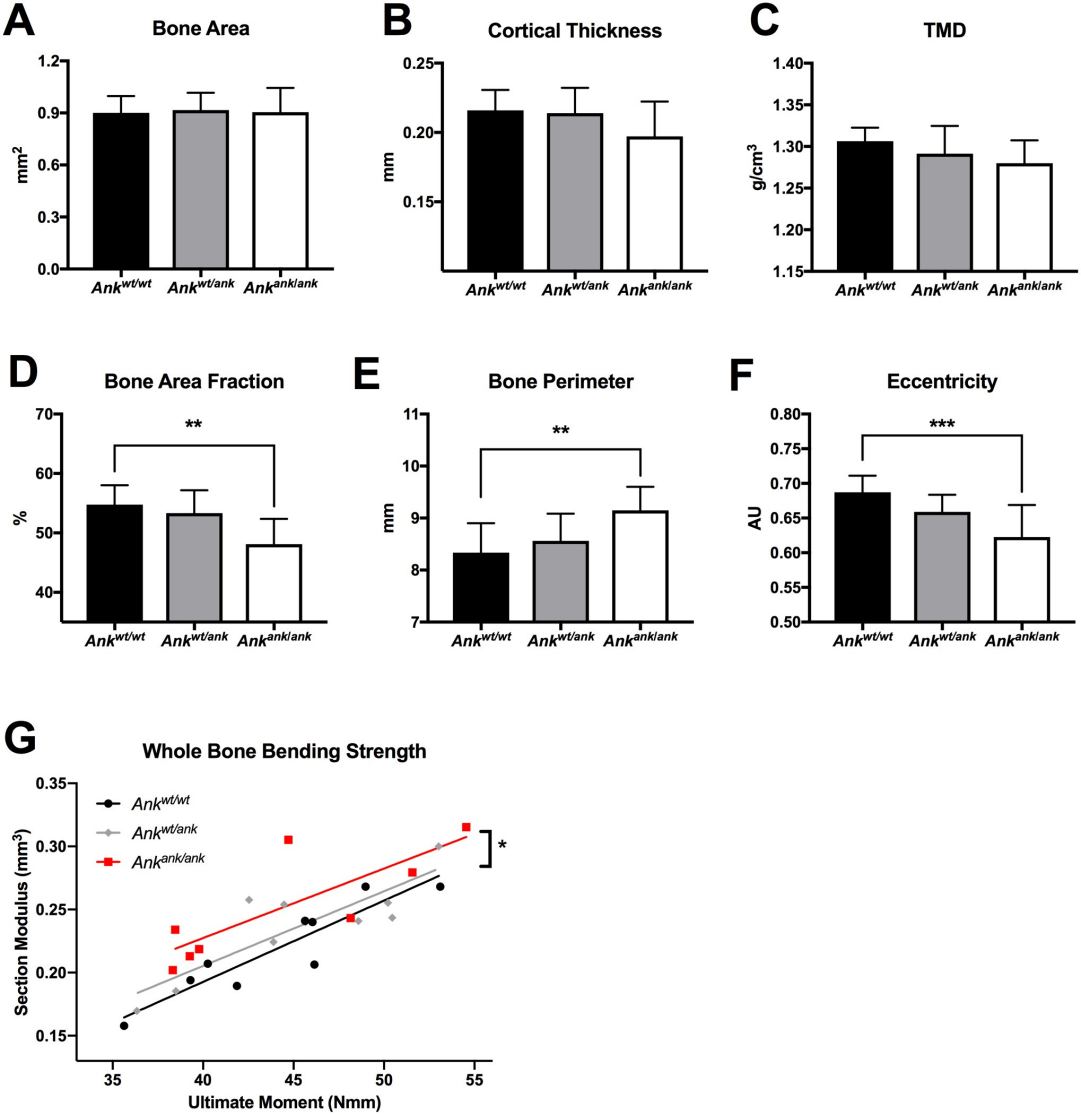
Bone geometry and mechanical performance is altered in the absence of ANK activity. microCT was used to determine (A) bone area, (B) cortical thickness, (C) tissue mineral density (TMD), (D) bone area fraction (B.Ar/T.Ar), (E) bone perimeter, and (F) eccentricity in femora of wild type (*Ank*^*wt/wt*^, n = 9), heterozygous (*Ank*^*wt/ank*^, n = 10) and *Ank*^*ank*/*ank*^ (n = 8) mice. (G) To compare whole bone bending strength, a linear regression between section modulus and ultimate bending moment was analyzed for each genotype (r^2^ = 0.84 *Ank*^*wt/wt*^, 0.73 *Ank*^*wt/ank*^, 0.67 *Ank*^*ank*/*ank*^). The slope was not different between genotypes, but the intercept was significantly different in femora from *Ank*^*ank*/*ank*^ mice, which utilized an increased section modulus to achieve the corresponding ultimate moment. The measurements presented in panels A-F, were obtained at the femoral midshaft, using an ROI of 1 mm. Statistical significance was determined by one-way ANOVA with * p < 0.05, ** p < 0.01, *** p < 0.001.

We next explored the consequences of the absence of Ank activity on bone physiology, by characterizing geometry and mineral density of femora harvested from *Ank*^*ank*/*ank*^, wild type and mice heterozygous for *ank* by microCT. At 3 months of age, most of the bone parameters, including bone area (Fig 6A), tissue mineral density (TMD, Fig 6B), and cortical thickness (Fig 6C), were not significantly different between wild type and *Ank*^*ank*/*ank*^ mice. However, significant differences in cortical bone properties between *Ank*^*ank*/*ank*^ and wild type mice were detected for bone area fraction (-12.1%), cortical bone perimeter (+9.8%), and cross-sectional geometry as indexed by eccentricity (-9.4%). Next, the structural and material properties of the bone were determined by standard three-point bending. Plotting ultimate bending moment against section modulus (Fig 6G) yielded linear relationships for each genotype (r^2^ = 0.84 *Ank*^*wt/wt*^, 0.73 *Ank*^*wt/ank*^, 0.67 *Ank*^*ank*/*ank*^) that did not significantly differ in slope (p = 0.88). However, we observed that femora from *Ank*^*ank*/*ank*^ mice required significantly less force per equivalent area of bone to break, as demonstrated by a significant difference in regression intercept (p = 0.0170). Taken together, our results indicate that the geometry of femora of *Ank*^*ank*/*ank*^ mice is altered and that these femora have diminished whole bone strength per equivalent amount of bone, results that are consistent with published data showing citrate deposition in bone affects hydroxyapatite nanostructure and strength [15].

## Discussion

ANKH is known for its important role in the prevention of pathological mineralization of joints, and its absence results in severe, progressive, ankylosis in both, humans and mice. It was previously thought that the main function of ANKH lies in regulation of extracellular PPi homeostasis, but here we identified a new and previously unanticipated function of ANKH: regulation of extracellular citrate concentrations. Although citrate has long been known to be a major compound in plasma, urine and bone, the mechanism used by cells to extrude citrate has been elusive. Our current data firmly link a specific membrane protein, ANKH, to extracellular citrate disposition *in vivo* and are in line with a GWAS study describing a correlation between plasma citrate levels and certain *ANKH* variants in humans [17]. Like citrate, succinate and malate are also present in plasma, though at substantially lower concentrations [18]. Our data showing that HEK293 cells overproducing ANKH release succinate and malate suggest ANKH also affects plasma levels of these Krebs-cycle intermediates. The very low concentrations of succinate in urine of a human patient lacking functional ANKH, further supports this hypothesis.

Extracellular citrate is present in many tissues and body fluids where it serves diverse and, in some cases, unknown functions [13]. In human plasma citrate levels are substantial, ranging from 60–100 μM in healthy individuals and can reach values of up to 300 μM in certain disease conditions [19,20]. Several tissues and cell types express citrate uptake transporters of the SLC13A family and plasma citrate plays important roles in (patho)physiology. Hepatic uptake of citrate from the circulation by SLC13A5 has, for instance, been implied in development of type 2 diabetes[21]. Intriguingly, extracellular citrate has been shown to alter cancer cell metabolism and cancer development [13]. Other functions of plasma citrate potentially include providing an energy source for cells under hypoglycemic conditions and acting as endogenous anticoagulant to prevent pathological blood clotting[13,22]. As ANKH affects plasma citrate concentrations, this membrane protein can be anticipated to affect the above-mentioned processes.

Via glomerular filtration, plasma citrate ends up in urine, where it reaches millimolar concentrations and prevents kidney stone formation [23]. Whereas urine of the *Ank*^*ank/ank*^ mice still contained substantial amounts of citrate, that of the human patient lacking functional ANKH was virtually devoid of citrate. This difference might be partly explained by dietary differences: Citrate has a high bioavailability of 80–90% [24] and is present in standard rodent food. Possibly, the human patient with biallelic inactivating mutations in ANKH had a diet that was low in citrate, whereas part of the citrate detected in plasma of *Ank*^*ank/ank*^ mice comes from dietary sources. Other factors potentially contributing to differences in urinary citrate excretion between humans and mice lacking functional ANKH/Ank are the differences in transport kinetics of the human and mouse citrate uptake transporters[25] and plasma acid-base status[26]. SLC13A5 is an uptake transporter crucially involved in citrate excretion, by reabsorbing citrate from primary urine. As only the divalent form of citrate is subject to SLC13A5-mediated transport, uptake is highly dependent on the pH of primary urine. The current view is that urinary excretion of citrate predominantly depends on plasma levels and SLC13A5-dependent reabsorption. Future studies are warranted to reveal if ANKH in the kidneys contributes to direct citrate release into urine.

Most of the body’s citrate, over 90%, is present in bone tissue [15]. Our results show that about 50% of bone citrate depends on Ank activity, in line with the high expression of *Ank* in osteoblasts [11]. The reduced strength of *Ank*^*ank/ank*^ bones, *i*.*e*. the altered relationship between ultimate moment and section modulus, is in line with the described role of citrate in stabilizing hydroxyapatite[15]. The altered eccentricity and perimeter of *Ank*^*ank*/*ank*^ femora are most likely a result of compensatory bone remodeling to retain whole bone strength. Interestingly, Ma *et al*. recently reported that local levels of extracellular citrate are important for the osteogenic development of human mesenchymal stem cells [16]. Kim et al showed that differentiation of osteoblast is delayed in mice lacking Ank, which was attributed to reduced extracellular levels of PPi [11]. This differentiation defect could be partly corrected in BMSCs (bone marrow-derived stem cells) by high, supra physiological, concentrations of extracellular PPi (500 μM). Our data indicate reduced levels of extracellular citrate at least partly underly the observed delay in osteogenic differentiation of *Ank*^*ank/ank*^ osteoblast.

A second important finding of the current study is that most of the PPi found in the extracellular environment of ANKH containing cells, originates from released NTPs, which are extracellularly converted into their respective NMP and PPi by ENPP1. This contradicts earlier work, proposing direct Ank-dependent cellular efflux of PPi being the main source of extracellular PPi [1]. Our conclusion that NTP release underlies most of the PPi detected in the extracellular environment of ANKH-containing cells is based on the following observations. First, *in vitro* experiments showed that the majority of PPi found in the culture medium of HEK293-*ANKH*^*wt*^ cells was derived from NTP efflux. Earlier work already indicated cells release ATP in an ANKH-dependent manner [27–29], but did not quantify relative amounts of extracellular ATP, AMP and PPi. It was therefore concluded that ATP was an ANKH substrate next to PPi. Convincing additional evidence arguing against direct PPi transport by Ank comes from our analysis of bones of mice lacking ENPP1 (*asj*^*GrsrJ*^ mice), which we found to be virtually devoid of PPi. If Ank would directly transport PPi, the 75% of PPi that ends up in bone via Ank (Fig 4) should not be affected by the absence of Enpp1. ENPP1 is abundantly present in bone tissue[30] allowing efficient conversion of NTPs released via ANKH into the bone matrix.

ABCC6 activity is responsible for ~70% of plasma PPi concentrations. [8,9]. *Ank*^*ank/ank*^ mice can therefore be anticipated to have at most a minor reduction (<30%) in plasma PPi concentrations. The dramatically reduced PPi levels in bones of the Ank mutant must therefore be a consequence of lack of local Ank-dependent PPi formation. These data indicate that ANKH has a crucial role in regulating local PPi homeostasis, whereas ABCC6 is the more important factor for keeping systemic PPi concentrations within the physiological range.

Absence of ANKH/Ank is associated with osteopenia of long bones in both, humans and mice [5,11]. It has been speculated that the bone phenotype seen upon ANKH loss originates from mechanical unloading [5]. Citrate is known to stabilize apatite nanocrystals [15] and mice lacking extracellular PPi suffer from severe osteopenia[31]. We hypothesize that Ank controls bone mineral density by affecting incorporation of citrate and PPi into hydroxyapatite. Such a function would fit data of previous studies showing that bones of *Enpp1*^*-/-*^ mice, which virtually lack PPi, have a substantially greater reduction in mineral density [32,33] than bones of *Ank*^*ank*/*ank*^ mice. These data also indicate that the residual 25% of PPi found in bones of *Ank*^*ank*/*ank*^ mice suffices to a large extent to keep BMD close to the normal range. Despite their different mechanism of action, the effects of PPi on mineral density are similar to the effects of bisphosphonates, pharmaceutical PPi analogues that are widely used in the treatment of osteoporosis [34]. Kim et al [11] have previously found a more dramatic effect of ANK on bone mineral density, a difference that might be explained by the different genetic background of their *Ank*^*ank*/*ank*^ mice.

Pyrophosphate in plasma is a crucial factor to prevent connective tissue mineralization [6]. Given that citrate concentrations in plasma depended on Ank activity, ANKH most likely also contributes to plasma PPi concentrations. Hepatic ABCC6-mediated ATP release underlies 60–70% of plasma PPi [8,9]. Ank can therefore be expected to be responsible for part of remaining 30–40% PPi present in plasma. The relatively small contribution of Ank together with the large variability in plasma PPi concentrations [8,9,35] prevents determination of the contribution of Ank to plasma PPi in *Ank*^*ank*/*ank*^ mice. Instead, *Ank*^*ank/ank*^;*Abcc6*^*-/-*^ compound mutant mice (*Ank*^*ank*^;*Abcc6*^*tm1Jfk*^) might be better suited to determine the contribution of ANKH/Ank to plasma PPi. In case ANKH substantially contributes to plasma PPi, it represents an attractive pharmacological target in the ectopic mineralization disorder pseudoxanthoma elasticum, which is caused by low plasma levels of PPi due to absence of functional ABCC6 [36,37].

Citrate might also contribute to the mineralization inhibitory effect of ANKH, as it strongly chelates calcium and is known to prevent kidney stone (uroliths) formation [23]. Notably, the observation of *Ho et al*. [1] that *Ank*^*ank*/*ank*^ mice have an increased incidence of kidney calcification would fit a function of Ank in prevention of ectopic mineralization in tissues different from those lining the joints. Possibly, also part of the ankylosis inhibitory effect of ANKH might come from citrate released into the joint space.

Our data clearly show that ANKH increases the abundance of NTPs and citrate in the extracellular environment. Theoretically, reduced cellular uptake and/or reduced extracellular degradation could also explain the increased levels of ATP and citrate in the extracellular environment. We consider these alternative explanations for the increased extracellular metabolite levels in medium of the HEK293-ANKH cells unlikely, however. First, reduced uptake cannot explain increased extracellular levels of ATP and other nucleotides, as mammalian cells do not have nucleotide uptake transporters. Second, although citrate uptake transporters have been described[13], they are hardly expressed by HEK293 cells (www.proteinatlas.org and [38]). Third, citrate is not metabolized extracellularly at considerable rates. Inhibition of degradation can therefore not account for the high levels of citrate in the culture medium.

In conclusion, we identified ANKH as an important player in the cellular release of citrate and NTPs that profoundly affects citrate and PPi disposition *in vivo* and is critical for normal bone development. Extracellular citrate is abundant, and ANKH is expressed in many tissues. We therefore expect our work to spur new lines of research exploring additional roles of ANKH and extracellular citrate in (patho)physiology.

## Materials and methods

### Ethics statement

Animal studies were approved by the Institutional Animal Care and Use Committee of Thomas Jefferson University in accordance with the National Institutes of Health Guide for Care and Use of Laboratory Animals under approval number 02081.

Urine analysis was approved by the MEC of Radboud University Medical Centre and consented by the patient carrying biallelic inactivating mutations in ANKH, and age-matched control [5].

### Cell culture

HEK293 cells were passaged in HyClone DMEM (GE) supplemented with 5% FBS and 100 units pen/strep per ml (Gibco) at 37^°^C and 5% CO_2_ under humidified conditions. Efflux experiments were performed in 6-well plates. 500,000 cells were seeded per well and 2 days later the experiment was started by replacing the culture medium with 2.5 ml Pro293a medium (Lonza), supplemented with 2 mM L-glutamine and 100 units pen/strep (Gibco) per ml. Samples were taken at the indicated time points. The presence of equal numbers of cells at the time of the experiment was confirmed by quantifying relative intracellular ATP levels per well (S2 Fig).

### Animals

Mice heterozygous for the progressive ankylosis allele (*ank*) were obtained from The Jackson Laboratory (Bar Harbor, ME; C3FeB6 *A/A*^*w-J*^*-Ank*^*ank/J*^, stock number 000200). Heterozygote breeders were used to generate *Ank*^*ank*/*ank*^, heterozygous and wild type littermates. Animals analyzed were between 11–14 weeks old. Plasma samples were collected by cardiac puncture in heparinized syringes. Studies included similar numbers of male and female mice.

### Mutagenesis and overexpression of ANKH

*ANKH*^*wt*^ cDNA was obtained from Sino Biological and subcloned into pEntr223 by USER cloning. The *L244S* mutation was introduced by USER cloning with primers 5’-ACCAGAAGCUCAGCATCTTTCTTATTGTTGCATCTCCC-3’ and AGCTTCTGGUGGCCTTCCGCTC TAATTCTGGCCACA. cDNAs were subsequently subcloned in a Gateway compatible pQCXIP expression vector [8]. HEK293 cells were transfected with pQCXIP-ANKH by calcium phosphate precipitation. ANKH^wt^ and ANKH^L244S^ in clones resistant to 2 μM puromycin were determined by immunoblot analysis, with a polyclonal antibody directed against ANKH (OAAB06341, Aviva Systems Biology). A mouse monoclonal antibody directed against the Na^+^K^+^-ATPase was used to show equal loading (S1 Fig).

### Enzymatic quantification of PP_i_, AMP and citrate

In medium samples, PP_i_ and AMP were quantified as described [9] with modifications. PPi concentrations were determined using ATP sulfurylase from NEB, and adenosine 5’phosphosulfate from Cayman Chemicals. AMP was quantified as follows: To 1 μl of sample or standard, 100 μl of a solution containing 0.14 U/ml pyruvate orthophosphate dikinase (PPDK, kind gift of Kikkoman Chemifa), 12.5 μmol/L PP_i_ (Sigma-Aldrich), 40 μmol/L phosphoenol pyruvate (Cayman Chemicals), 50 μmol/L dithiothreitol, 1 mmol/L EDTA, 7.5 mmol/L MgSO_4_ and 30 mmol/L BES (pH 8.0) was added. Conversion of AMP into ATP was allowed to proceed for 20 min at 45 °C, after which PPDK was inactivated by incubation at 80 °C for 10 min. To determine PPi and citrate amounts in bones, tibiae and femora of 13-week-old mice were collected and defleshed. Epiphyses were removed and bone marrow was spun out of the bones (30,000 RCF, 1 min). Bones were subsequently dissolved by incubation with continuous mixing in 10% formic acid (60 °C, 750 RPM, 14 hrs). Samples were spun for 10 min at 30,000 RCF and the supernatant was analyzed for PPi and citrate content. For bone extracts a slightly modified, more sensitive, version of the PPi assay was used. A total reaction volume of 520 μl assay mix contained 100 μl of SL-ATP detection reagent (Biothema, Sweden), 0.1 μl ATP removal reagent (“apyrase”, BioThema, Sweden), 6 μM adenosine-5’-phosphosulphate (APS) (SantaCruz, TX), 0.15 U/ml ATP sulphurylase (ATPS) (New England Biolabs) and 400 μl of ATP-free Tris-EDTA buffer (BioThema, Sweden) was first incubated overnight at room temperature to convert PPi into ATP for subsequent degradation by apyrase. The overnight incubation removed background PPi from the assay mixture, resulting in a higher sensitivity of the assay. Next, the sample, diluted 500-fold in Tris-EDTA buffer, was added to 500 μl of the assay mixture, resulting in an increase in luminescence due to the conversion of PPi and APS into ATP, a reaction catalyzed by ATPS. Finally, a known amount of ATP was added as internal standard and the ratio between the increase in bioluminescent signal induced by the addition of PPi and by the increase induced by the addition of ATP was used to calculate the PPi concentration. The assay was performed in a Berthold FB12 luminometer in the linear range of the detector. Internal PPi standards were used to show robustness and sensitivity of the assay.

Citrate was quantified in medium samples using the Megazyme Citric Acid Kit (Megazyme, Ireland).

### Real-time ATP efflux assay

Real-time ATP efflux assays were performed as described [9], with modifications. To reduce ATP release by the initial buffer change, cells were incubated at 27°C, for 1 hr. Then an additional 50 μl of ATP efflux buffer containing 10% of ATP-monitoring reagent (BactiterGlo, Promega), dissolved in ATP efflux buffer was added. Bioluminescence was followed in real-time for 1 hr at 27 **°**C and 2 hrs at 37 **°**C in a Flex Station3 microplate reader (Molecular Devices).

### LC/MS-based global metabolite profiling

Proteins were precipitated in 200 μl of medium or 50 μl plasma by adding 800 μl and 200 μl acetonitrile:methanol (1:1), respectively. Samples were shaken (10 minutes, 500 RPM, 21°C), centrifuged (15,000 g, 4°C, 10 min) and the supernatant dried in a Speed-Vac. Pellets were stored at -20°C until analysis. For analysis pellets were suspended in 45 μl mobile phase A of which 10 μl was analyzed by ion-pairing LC/MS as described [10].

Analytes were identified based on accurate mass and retention time, which matched reference standards. Peak areas were determined using Masshunter Qualitative Analysis software version 7.0SP2 (Agilent Technologies).

### LC/MS-based quantification of citrate

Plasma proteins were removed as described above and resuspended in 50 ul mobile phase A, while urine and bone samples were diluted in mobile phase A (5 and 20-fold, respectively). A volume of 5 μl of each sample was analyzed as described under LC/MS global metabolite profiling, along with calibration curves consisting of mobile phase A spiked with citrate concentrations ranging from 1 to 1000 μM. Quantification was performed using Masshunter Profinder Quantitative Analysis software version B.08.00, service pack 3 (Agilent Technologies).

### NMR spectroscopy

One-dimensional ^1^H-NMR spectroscopy of urine samples was performed as described [39]. Briefly, urine samples were centrifuged for 10 min at 3,000 g and trimethylsilyl-2,2,3,3-tetradeuteropropionic acid (TSP; sodium salt; Sigma) in D_2_O was added before analysis to serve both, as an internal quantity reference and a chemical shift reference. The pH of each sample was adjusted to 2.50 ± 0.05 with concentrated HCl. ^1^H-NMR spectra were obtained using a Bruker 500-MHz spectrometer (pulse angle: 90°; delay time: 4 s; no. of scans: 256; relaxation delay: 2s). Assignment of peak positions for compound identification was performed by comparing the peak positions in the spectra of the metabolites with the reference spectral database of model compounds at pH 2.5 using Amix version 3.9.14 (Bruker BioSpin).

### Calculation of the contribution of NTP release to ANKH-dependent accumulation of PPi in the culture medium

To estimate the contribution of ANKH^wt^-mediated NTP release to 24-hour extracellular PPi concentrations, PPi concentrations in medium of HEK293 parental cells were subtracted from the PPi concentrations detected in medium of HEK293-*ANKH*^*wt*^ cells, yielding an ANKH-specific PPi accumulation in the 24-hr culture medium samples of 2.4 μM. The same calculation demonstrated an ANKH-specific accumulation of 1.4 μM AMP in the culture medium. This demonstrated that ATP release underlies at least 60% of the ANKH-dependent PPi accumulation detected in the culture medium (1.4/2.4 x 100 = 58). GMP, UMP and CMP were also found to increase in culture medium in an ANKH-dependent manner. Based on the relative LC/MS signals of the NMPs, we estimated that AMP was responsible for 80% of the total NMP concentration in the culture medium, whereas GMP, UMP and CMP together were responsible for the remaining 20%. Together these data demonstrate that nucleoside monophosphate (NMP) concentrations could explain 70% of the ANKH-dependent PPi that had accumulated in the culture medium after 24 hrs. The calculated 70% is most likely an underestimation, as generated NMPs will be further metabolized by the HEK293 cells, as demonstrated by our previous work [8]. Further metabolism of AMP also explains the discordance between AMP and PPi concentrations in the culture medium.

### MicroCT

Each bone was scanned using a Bruker Skyscan 1275 microCT system equipped with a 1 mm aluminum filter. One femur from each mouse was scanned at 55 kV and 181 μA with a 74 ms exposure time. Transverse scan slices were obtained by placing the long axis of the bone parallel to the z axis of the scanner using a custom 3D printed sample holder. An isometric voxel size of 13 μm was used. Images were reconstructed using nRecon (Bruker) and analyzed using CTan (Bruker). Cortical bone was analyzed using a 1 mm thick region of interest centered at the mid-diaphysis of the femur. Quantitative analysis was performed in accordance with the recommendations of the American Society for Bone and Mineral Research [40].

### Three-point bending assay

Three-point bending was performed on bones that had been stored at -20 °C in PBS-soaked gauze after harvest. Femora were scanned with microCT before performing three-point bending. Briefly, each femur was oriented on a standard fixture with femoral condyles facing down and a bending span of 8.7 mm. Next, a monotonic displacement ramp of 0.1 mm/s was applied until failure, with force and displacement acquired digitally. The force-displacement curves were converted to stress-strain using microCT-based geometry and analyzed using a custom GNU Octave script.

### Statistical analyses

P-values of group comparisons were calculated using one-way Anova using Prism 7.0d version (GraphPad Software Inc.), unless otherwise indicated in the figure legends. Significance is indicated in the figures, with * < 0.05, ** < 0.01, *** < 0.001 and **** < 0.0001.

## Supporting information

S1 Fig Detection of ANKH in HEK293 cells overproducing ANKH^wt^ or ANKH^L244S^ using rabbit anti-ANKH (C-terminal region, OAAB06341, Aviva Systems Biology). Mouse anti-Na^+^/K^+^-ATPase (ab7671, ABCAM) was used as a loading control. Boxed areas: approximate region of blot presented in panel A of Fig 1. ^1)^ membrane fraction of HEK293-ANKHwt clone C3. ^2)^ membrane fraction of HEK293 parental cells.
(PDF)

S2 Fig Relative amounts of ATP determined by LC/MS in cell pellets of HEK293 parental, HEK293-*ANKH*^*wt*^ and HEK293-*ANKH*^*L244S*^ cells grown in wells of a 6-well plate as described in the materials and methods section. Data represent mean +/- SD of an experiment performed in triplicate.
(PDF)

S3 Fig The L244S mutation completely inactivates ANKH as determined by following extracellular PPi levels (A) and citrate release (B) in culture medium of an independent HEK293 clone (B1) that stably overproduces ANKH^L244S^. Concentrations of PPi and citrate were determined in 24-hour medium samples of parental HEK293 cells, HEK293 cells stably overproducing ANKH^wt^ (clone C3) and HEK293 cells overproducing ANKH^L244S^ (clone B1). Statistical significance was determined by ANOVA with Tukey correction. *** p < 0.001; **** p < 0.0001, ANKH^wt^ C3 vs parental.
(PDF)

## References

[1] Ho AM, Johnson MD, Kingsley DM. Role of the mouse ank gene in control of tissue calcification and arthritis. Science. 2000;289: 265–270. 10.1126/science.289.5477.265 10894769

[2] Kawasaki K, Buchanan AV, Weiss KM. Biomineralization in humans: making the hard choices in life. Annu Rev Genet. 2009;43: 119–142. 10.1146/annurev-genet-102108-134242 19659443

[3] Gurley KA, Chen H, Guenther C, Nguyen ET, Rountree RB, Schoor M, et al Mineral formation in joints caused by complete or joint-specific loss of ANK function. J Bone Miner Res. 2006;21: 1238–1247. 10.1359/jbmr.060515 16869722

[4] Abhishek A, Doherty M. Pathophysiology of articular chondrocalcinosis—role of ANKH. Nat Rev Rheumatol. 2011;7: 96–104. 10.1038/nrrheum.2010.182 21102543

[5] Morava E, Kühnisch J, Drijvers JM, Robben JH, Cremers C, van Setten P, et al Autosomal recessive mental retardation, deafness, ankylosis, and mild hypophosphatemia associated with a novel ANKH mutation in a consanguineous family. J Clin Endocrinol Metab. 2011;96: E189–98. 10.1210/jc.2010-1539 20943778 PMC5393418

[6] Orriss IR, Arnett TR, Russell RGG. Pyrophosphate: a key inhibitor of mineralisation. Curr Opin Pharmacol. 2016;28: 57–68. 10.1016/j.coph.2016.03.003 27061894

[7] Nitschke Y, Rutsch F. Inherited Arterial Calcification Syndromes: Etiologies and Treatment Concepts. Curr Osteoporos Rep. 2017;15: 1–16. 10.1007/s11914-017-0341-8 28585220

[8] Jansen RS, Küçükosmanoglu A, de Haas M, Sapthu S, Otero JA, Hegman IEM, et al ABCC6 prevents ectopic mineralization seen in pseudoxanthoma elasticum by inducing cellular nucleotide release. Proc Natl Acad Sci USA. 2013;110: 20206–20211. 10.1073/pnas.1319582110 24277820 PMC3864344

[9] Jansen RS, Duijst S, Mahakena S, Sommer D, Szeri F, Váradi A, et al ABCC6-mediated ATP secretion by the liver is the main source of the mineralization inhibitor inorganic pyrophosphate in the systemic circulation-brief report. Arterioscler Thromb Vasc Biol 2014;34: 1985–1989. 10.1161/ATVBAHA.114.304017 24969777 PMC6743317

[10] Goncalves MD, Lu C, Tutnauer J, Hartman TE, Hwang S-K, Murphy CJ, et al High-fructose corn syrup enhances intestinal tumor growth in mice. Science. 2019;363: 1345–1349. 10.1126/science.aat8515 30898933 PMC6487857

[11] Kim HJ, Minashima T, McCarthy EF, Winkles JA, Kirsch T. Progressive ankylosis protein (ANK) in osteoblasts and osteoclasts controls bone formation and bone remodeling. J Bone Miner Res. 2010;25: 1771–1783. 10.1002/jbmr.60 20200976 PMC3153348

[12] Alfrey AC, Solomons CC. Bone pyrophosphate in uremia and its association with extraosseous calcification. J Clin Invest. 1976;57: 700–705. 10.1172/JCI108327 175092 PMC436704

[13] Mycielska ME, Milenkovic VM, Wetzel CH, Rümmele P, Geissler EK. Extracellular Citrate in Health and Disease. Curr Mol Med. 2015;15: 884–891. 10.2174/1566524016666151123104855 26592250

[14] Petrarulo M, Facchini P, Cerelli E, Marangella M, Linari F. Citrate in urine determined with a new citrate lyase method. Clin Chem. 1995;41: 1518–1521. 7586527

[15] Hu Y-Y, Rawal A, Schmidt-Rohr K. Strongly bound citrate stabilizes the apatite nanocrystals in bone. Proc Natl Acad Sci USA. 2010;107: 22425–22429. 10.1073/pnas.1009219107 21127269 PMC3012505

[16] Ma C, Tian X, Kim JP, Xie D, Ao X, Shan D, et al Citrate-based materials fuel human stem cells by metabonegenic regulation. Proc Natl Acad Sci USA. 2018;115: E11741–E11750. 10.1073/pnas.1813000115 30478052 PMC6294936

[17] Shin S-Y, Fauman EB, Petersen A-K, Krumsiek J, Santos R, Huang J, et al An atlas of genetic influences on human blood metabolites. Nat Genet. 2014;46: 543–550. 10.1038/ng.2982 24816252 PMC4064254

[18] Klein MS, Almstetter MF, Nürnberger N, Sigl G, Gronwald W, Wiedemann S, et al Correlations between milk and plasma levels of amino and carboxylic acids in dairy cows. J Proteome Res. 2013;12: 5223–5232. 10.1021/pr4006537 23931703

[19] Iacobazzi V, Infantino V. Citrate—new functions for an old metabolite. Biol Chem. 2014;395: 387–399. 10.1515/hsz-2013-0271 24445237

[20] Rudman D, Dedonis JL, Fountain MT, Chandler JB, Gerron GG, Fleming GA, et al Hypocitraturia in patients with gastrointestinal malabsorption. N Engl J Med. 1980;303: 657–661. 10.1056/NEJM198009183031201 7402252

[21] Birkenfeld AL, Lee H-Y, Guebre-Egziabher F, Alves TC, Jurczak MJ, Jornayvaz FR, et al Deletion of the mammalian INDY homolog mimics aspects of dietary restriction and protects against adiposity and insulin resistance in mice. Cell Metabolism. 2011;14: 184–195. 10.1016/j.cmet.2011.06.009 21803289 PMC3163140

[22] Mycielska ME, Sun H, Dettmer K, Rümmele P, Schmidt K, Prehn C, et al Extracellular Citrate Affects Critical Elements of Cancer Cell Metabolism and Supports Cancer Development In Vivo. Cancer Res. 2018;78: 2513–2523. 10.1158/0008-5472.CAN-17-2959 29510993

[23] Raffin EP, Penniston KL, Antonelli JA, Viprakasit DP, Averch TD, Bird VG, et al The Effect of Thiazide and Potassium Citrate Use on the Health Related Quality of Life of Patients with Urolithiasis. J Urol. 2018;200: 1290–1294. 10.1016/j.juro.2018.06.023 29913138

[24] Harvey JA, Zobitz MM, Pak CY. Bioavailability of citrate from two different preparations of potassium citrate. J Clin Pharmacol. 1989;29: 338–341. 10.1002/j.1552-4604.1989.tb03338.x 2723122

[25] Pajor AM. Sodium-coupled dicarboxylate and citrate transporters from the SLC13 family. Pflugers Arch. 2014;466: 119–130. 10.1007/s00424-013-1369-y 24114175

[26] Goraya N, Simoni J, Sager LN, Madias NE, Wesson DE. Urine citrate excretion as a marker of acid retention in patients with chronic kidney disease without overt metabolic acidosis. Kidney Int. 2019;95: 1190–1196. 10.1016/j.kint.2018.11.033 30846270

[27] Rosenthal AK, Gohr CM, Mitton-Fitzgerald E, Lutz MK, Dubyak GR, Ryan LM. The progressive ankylosis gene product ANK regulates extracellular ATP levels in primary articular chondrocytes. Arthritis Res Ther. 2013;15: R154 10.1186/ar4337 24286344 PMC3978574

[28] Costello JC, Rosenthal AK, Kurup IV, Masuda I, Medhora M, Ryan LM. Parallel regulation of extracellular ATP and inorganic pyrophosphate: roles of growth factors, transduction modulators, and ANK. Connect Tissue Res. 2011;52: 139–146. 10.3109/03008207.2010.491928 20604715

[29] Mitton-Fitzgerald E, Gohr CM, Bettendorf B, Rosenthal AK. The Role of ANK in Calcium Pyrophosphate Deposition Disease. Curr Rheumatol Rep. 2016;18: 25 10.1007/s11926-016-0574-z 27032788 PMC5453179

[30] Kato K, Nishimasu H, Okudaira S, Mihara E, Ishitani R, Takagi J, et al Crystal structure of Enpp1, an extracellular glycoprotein involved in bone mineralization and insulin signaling. Proc Natl Acad Sci USA. 2012;109: 16876–16881. 10.1073/pnas.1208017109 23027977 PMC3479499

[31] Huesa C, Staines KA, Millán JL, MacRae VE. Effects of etidronate on the Enpp1^−^/^−^ mouse model of generalized arterial calcification of infancy. Int J Mol Med. 2015;36: 159–165. 10.3892/ijmm.2015.2212 25975272 PMC4494596

[32] Albright RA, Stabach P, Cao W, Kavanagh D, Mullen I, Braddock AA, et al ENPP1-Fc prevents mortality and vascular calcifications in rodent model of generalized arterial calcification of infancy. Nature Commun. 2015;6: 10006 10.1038/ncomms10006 26624227 PMC4686714

[33] Li Q, Guo H, Chou DW, Berndt A, Sundberg JP, Uitto J. Mutant Enpp1asj mice as a model for generalized arterial calcification of infancy. Dis Models Mech. 2013;6: 1227–1235. 10.1242/dmm.012765 23798568 PMC3759342

[34] Russell RGG. Bisphosphonates: From Bench to Bedside. Ann N Y Acad Sci. 2006;1068: 367–401. 10.1196/annals.1346.041 16831938

[35] Dedinszki D, Szeri F, Kozák E, Pomozi V, Tőkési N, Mezei TR, et al Oral administration of pyrophosphate inhibits connective tissue calcification. EMBO Mol Med. 2017;9: 1463–1470. 10.15252/emmm.201707532 28701330 PMC5666306

[36] Uitto J, Li Q, van de Wetering K, Váradi A, Terry SF. Insights into Pathomechanisms and Treatment Development in Heritable Ectopic Mineralization Disorders: Summary of the PXE International Biennial Research Symposium-2016. J Invest Dermatol. 2017;137: 790–795. 10.1016/j.jid.2016.12.014 28340679 PMC5831331

[37] Borst P, Váradi A, van de Wetering K. PXE, a Mysterious Inborn Error Clarified. Trends Biochem Sci. 2019;44: 125–140. 10.1016/j.tibs.2018.10.005 30446375 PMC6340748

[38] Mazurek MP, Prasad PD, Gopal E, Fraser SP, Bolt L, Rizaner N, et al Molecular origin of plasma membrane citrate transporter in human prostate epithelial cells. EMBO reports. EMBO Press; 2010;11: 431–437. 10.1038/embor.2010.51 20448665 PMC2892322

[39] Engelke UFH, Tassini M, Hayek J, de Vries M, Bilos A, Vivi A, et al Guanidinoacetate methyltransferase (GAMT) deficiency diagnosed by proton NMR spectroscopy of body fluids. NMR Biomed. 2009;22: 538–544. 10.1002/nbm.1367 19288536

[40] Bouxsein ML, Boyd SK, Christiansen BA, Guldberg RE, Jepsen KJ, Müller R. Guidelines for assessment of bone microstructure in rodents using micro-computed tomography. J Bone Miner Res. 2010;25: 1468–1486. 10.1002/jbmr.141 20533309

