# Supplementary material for: The membrane protein ANKH is crucial for bone mechanical performance by mediating cellular export of citrate and ATP
Source: PLoS Genet. 2020 Jul 8;16(7):e1008884. doi: 10.1371/journal.pgen.1008884 (PMC7371198; doi:10.1371/journal.pgen.1008884)
Supplement: S1 Fig — Mouse anti-Na+/K+-ATPase (ab7671, ABCAM) was used as a loading control. Boxed areas: approximate region of blot presented in panel A of Fig 1. 1) membrane fraction of HEK293-ANKHwt clone C3. 2) membrane fraction of HEK293 parental cells. (PDF) [file pgen.1008884.s001.pdf]

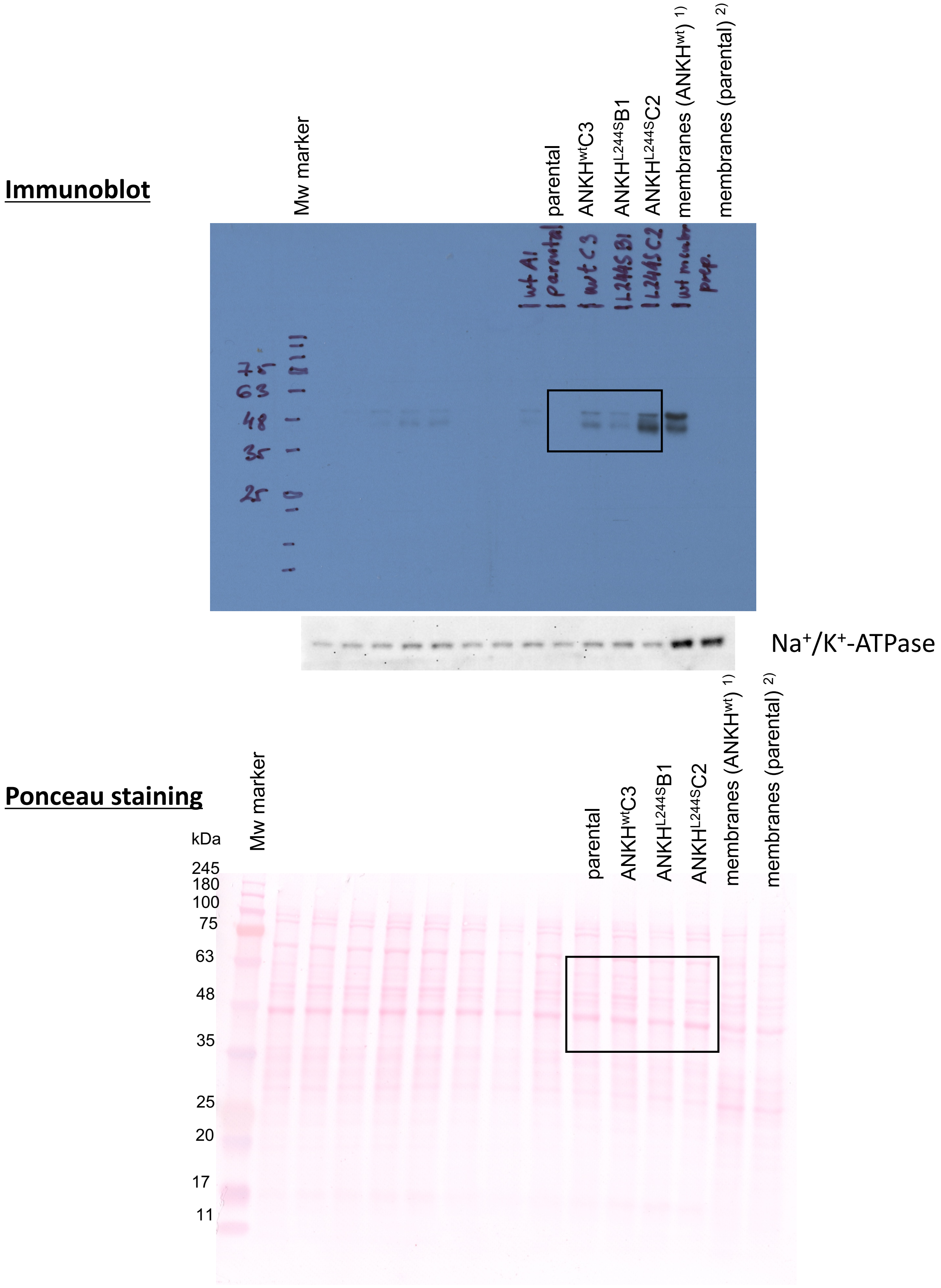

S1 Fig. Detection of ANKH in HEK293 cells overproducing ANKH^{wt} or ANKH^{L244S} using rabbit anti-ANKH (C-terminal region, OAAB06341, Aviva Systems Biology). Mouse anti-Na^+/K^+-ATPase (ab7671, ABCAM) was used as a loading control. Boxed areas: approximate region of blot presented in panel A of Figure 1. [1)] membrane fraction of HEK293-ANKHwt clone C3. [2)] membrane fraction of HEK293 parental cells
