# Supplementary material for: The membrane protein ANKH is crucial for bone mechanical performance by mediating cellular export of citrate and ATP
Source: PLoS Genet. 2020 Jul 8;16(7):e1008884. doi: 10.1371/journal.pgen.1008884 (PMC7371198; doi:10.1371/journal.pgen.1008884)
Supplement: S2 Fig — Data represent mean +/- SD of an experiment performed in triplicate. (PDF) [file pgen.1008884.s002.pdf]

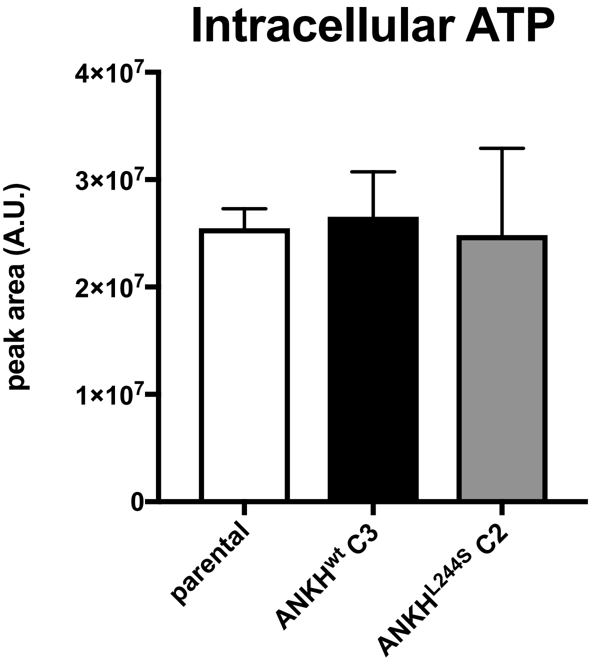

S2_Fig. Relative amounts of ATP determined by LC/MS in cell pellets of HEK293 parental, HEK293-*ANKH*^{wt} and HEK293-*ANKH*^{L244S} cells grown in wells of a 6-well plate as described in the materials and methods section. Data represent mean +/- SD of an experiment performed in triplicate.
