# Supplementary material for: The membrane protein ANKH is crucial for bone mechanical performance by mediating cellular export of citrate and ATP
Source: PLoS Genet. 2020 Jul 8;16(7):e1008884. doi: 10.1371/journal.pgen.1008884 (PMC7371198; doi:10.1371/journal.pgen.1008884)
Supplement: S3 Fig — Concentrations of PPi and citrate were determined in 24-hour medium samples of parental HEK293 cells, HEK293 cells stably overproducing ANKHwt (clone C3) and HEK293 cells overproducing ANKHL244S (clone B1). Statistical significance was determined by ANOVA with Tukey correction. *** p < 0.001; **** p < 0.0001, ANKHwt C3 vs parental. (PDF) [file pgen.1008884.s003.pdf]

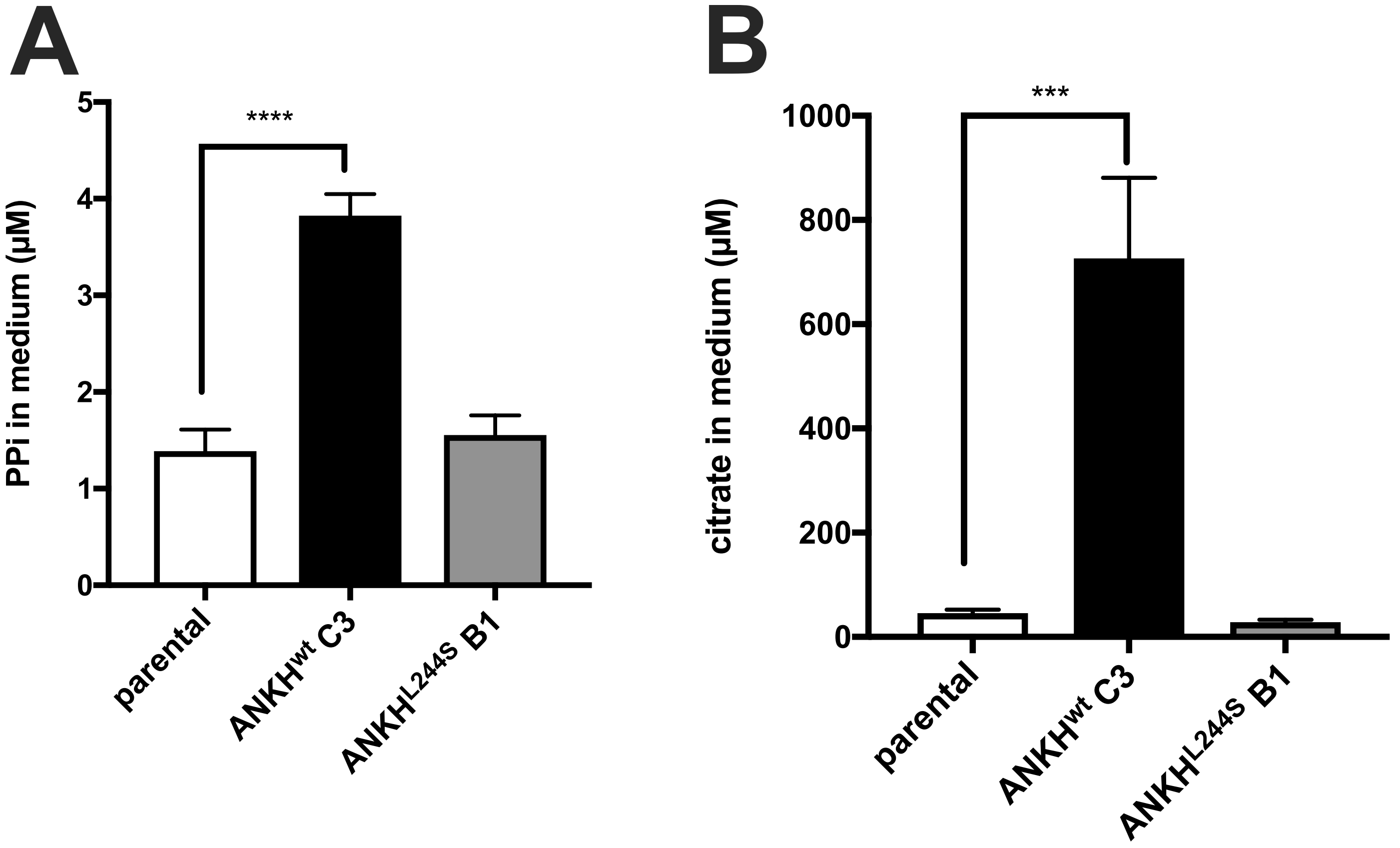

S3 Fig. The L244S mutation completely inactivates ANKH as determined by following extracellullar PPi (**A**) and citrate release (**B)** in culture medium by an independent HEK293 clone (B1) that stably overproduces ANKH^{L244S}. Concentrations of PPi and citrate were determined in 24-hour medium samples of parental HEK293 cells, HEK293 cells stably overproducing ANKH^{wt} (clone C3) and HEK293 cells overproducing ANKH^{L244S} (clone B1). Statistical significance was determined by ANOVA with Tukey correction. *** $p < 0.001$; **** $p < 0.0001$, ANKH^{wt} C3 vs parental.
