# Supplementary material for: The membrane protein ANKH is crucial for bone mechanical performance by mediating cellular export of citrate and ATP
Source: PLoS Genet. 2020 Jul 8;16(7):e1008884. doi: 10.1371/journal.pgen.1008884 (PMC7371198; doi:10.1371/journal.pgen.1008884)
Supplement: Attachment — Submitted filename: 200319_Review_Plos_Genetics_ANKH_citrate.docx [file pgen.1008884.s004.docx]

**Review Plos Genetics**

**PGENETICS-D-20-00369**

**The membrane protein ANKH is critical for normal development and plays a key role in the cellular export of citrate and ATP from mammalian cells**

**Summary**

The presented manuscript describes metabolic abnormalities of malate/citrate and nucleotide metabolism upon ANKH deactivation/mutation. The experiments were performed *in vitro* and *in vivo* analyzing stably transfected Hek293 cells and *Ank^ank/ank^* mice, respectively. In addition, urine samples of a CMD patient were analyzed for metabolic abnormalities. The authors describe increased extracellular levels of ATP and its degradation products AMP and PPi upon overexpression of ANKH_wt but not the mutant ANKH_L244S. This suggests that ANKH does not primarily regulates extracellular PPi levels but its progenitor metabolite ATP. Metabolite screening revealed an identical pattern for CMP, GMP, and UMP. In addition, the authors show increased extracellular citrate, malate, and succinate levels upon ANKH_wt but not ANKH_L244S overexpression. In bone tissue of *Ank^ank/ank^* mice the authors show reduced relative PPi content in mineralized bone tissue (femur, tibia). In addition, *Ank^ank/ank^* mice, lacking functional Ank protein, reveal reduced citrate levels in plasma, urine, and bone tissue. A CMD patient also showed reduced citrate and succinate levels. Next, femoral bone tissue of *Ank^ank/ank^* mice was morphologically investigated revealing alteration in selected parameters. Lastly, the whole long bone bending strength was assessed

**Major Critisism**

The manuscript is well written, the data are clearly presented, and potentially of relevance. However, the study does not convincingly set the findings in the complex context of bone biology, ankylosing spondylitis and nucleotide metabolism.

The authors do not comment that the manuscript has already been published by the BioRxiv server. doi: https://doi.org/10.1101/2019.12.20.883223

The title of the study implicated that human ANKH is critical for “normal development” and regulates “cellular export of citrate and ATP”. Both claims were not fully demonstrated in the study. PPi and citrate content was demonstrated in bone, plasma and urine of *Ank^ank/ank^* mice at a time point of 11-14 weeks but not during development at different stages. The study shows altered levels in vitro and in vivo after genetic manipulation of *ANKH* or *Ank*, respectively. The cellular model (Hek293 cells) is not appropriated to model developmental processes. The metabolic measurement of PPi, ATP, AMP, CMP, GMP, UMP, citrate, malate, and succinate appears overall fine. However, the authors cannot differentiate between cellular export, extracellular degradation, cellular import/internalization mechanisms. The authors do not address the cell surface levels/activity of nucleotide degradative or regulatory proteins (APs, ENNPs, ABCC, others etc).

The finding of increased extracellular levels of the major metabolite citrate is of high interest. It suggests that ANKH directly or indirectly affects the systemic citrate levels. This suggests a role of citrate for bone metabolism or mirrors simply the metabolic status of the entire organism. The reviewer assumes that the measurements reflect a systemic metabolic status, which indicates an impact of ANKH on metabolic processes specifically in non-mineralized tissues. For instance, the expression and subcellular localization of SLC13A5 or SLC25A1, two major citrate transporting proteins, should be addressed. Both proteins have significant species specificity (PMID 28264506, 20686672), thus, conclusions between mice and humans should be carefully considered.

The measured bone parameter in *Ank^ank/ank^* mice do not specify were the measurements have been done and the authors should follow the ASBMR nomenclature guidelines for bone measurements (PMID 23197339, 3455637). The bone analysis is not state of the art. The authors should set their long bone related findings in the context of the literature related to the *Ank^ank/ank^* bone phenotype (e.g. PMID 15833911, 20200976, 20943778). At the used age, *Ank^ank/ank^* mice have established the full ankylosing spondylitis phenotype. The authors ignore this aspect.

The mode of action of bisphosphonates is the generation of non-hydrolysable nucleotides inducing generalized perturbation of energy metabolism and protein lipid anchoring (prenylation). Due to the extreme enrichment at bone surfaces bisphosphonates mainly affect osteoclasts (21555003). Thus, the biological role of PPi and bisphosphonates should not mixed up!

The discussion of citrate and ANKH in other conditions (mental retardation, sperm formation) is speculative.

Parts of the conclusion are speculations that are not supported by the data basis and do not progress the understanding of ANKH function. *“In conclusion, we identified ANKH/ANK as an important player in the cellular release of citrate and NTPs that profoundly affects citrate and PPi disposition in vivo and is critical for normal bone development. Our data indicates extracellular citrate might have previously unanticipated roles in cognitive function and prevention of soft tissue mineralization [33,34]. We expect our work to spur new lines of research exploring additional roles of ANKH and extracellular citrate in (patho)physiology.”*

**Technical Criticism**

Fig1A. The shown Western blot is not very convincing. The reviewer is aware that detection of ANKH is difficult. Loading control of reference proteins is missing. Quantitative PCR would be important to verify ANKH transcript levels in the generated cell lines.

Fig1B-D. The shown PPi, AMP and RLU (ATP release) levels are not normalized to cell/protein content. This is not acceptable for two reasons: i) normalization for comparable cell seeding and ii) impact of proliferation specifically for long time ranges (Fig1B-C) was not considered. The authors do not comment on differences in proliferation but normalization of metabolites is critical.

Fig2A-C. Metabolic measurements have not been normalized to cell number or protein level. Please show values of cell line ANKHL244S B1.

Fig3A-C. Metabolic measurements have not been normalized to cell number or protein level. Please show values of cell line ANKHL244S B1.

Fig5C. The patient used for analysis is not a CMD patient. The ANKH variant p.L244S is a special mutation showing high homology with sign of *Ank^ank/ank^* mice (PMID 20943778).

Fig6A-F. Show clearly where the measurements have been done (site of bone). microCT should easily provide the images to demonstrate overall bone structure and used ROI.

Fig6G. The analysis reveals the overall bone strength. This type of analysis is not appropriated to differentiate the impact of composite bone components collagen, ECM, and mineral phase. Conclusions from this analysis regarding bone tissue function are not justified.

**Minor Critisism**

The used gene/protein nomenclature should be consistently according to Ensemble and NCBI. *Ank* - mouse gene, Ank - mouse protein, *ANKH* - human gene, ANKH - human protein. This makes reading easier. ANKH/ANK is not necessary.

For the mouse model used I would use a consistent nomenclature as well. For instance, *Ank^wt/wt^, Ank^wt/ank^, Ank^ank/ank^*.

The authors reference to the unpublished manuscript *Szeri et al*. Please reference only when published.
