# Supplementary material for: The membrane protein ANKH is crucial for bone mechanical performance by mediating cellular export of citrate and ATP
Source: PLoS Genet. 2020 Jul 8;16(7):e1008884. doi: 10.1371/journal.pgen.1008884 (PMC7371198; doi:10.1371/journal.pgen.1008884)
Supplement: Attachment — Submitted filename: Response to reviewers.docx [file pgen.1008884.s005.docx]

**Reviewer 1:**

**Major criticism**

1. **The title of the study implicated that human ANKH is critical for “normal development” and regulates “cellular export of citrate and ATP”. Both claims were not fully demonstrated in the study.**

We agree with reviewer 1 and changed the title to “**The membrane protein** **ANKH affects bone mechanical performance by controlling cellular citrate and ATP release**”**.**

We apologize for not clearly explaining why we come to the conclusion that ANKH is involved in release of NTPs and citrate. The increased levels of these metabolites in the culture medium of ANKH-containing HEK293 cells cannot be explained by reduced cellular uptake or reduced extracellular degradation for the following reasons:

a. Several metabolites accumulate in the culture medium of ANKH containing HEK293 cells. We specifically introduced ANKH in these HEK293 cells and it is unlikely that secondary downregulation of uptake transporters accounts for increases in extracellular levels of all these metabolites.
b. Both AMP and PPi go up, strongly indicating that ATP is being released by the cells.
c. Moreover, to the best of our knowledge, there are no uptake transporters known for ATP or AMP. Reduced uptake of these compounds can therefore not be responsible for the accumulation of AMP, GMP, UMP and CMP in the culture medium.
d. Theoretically, decreased extracellular metabolism could account for the increased levels of AMP and other NMPs in the culture medium. However, it cannot account for the increased levels of PPi detected in the culture medium of ANKH-containing cells, as TNAP, the major enzyme involved in PPi degradation, is not expressed by the parental HEK293 cells (proteinatlas.org and our unpublished data).
e. SLC13A1, A2 and A3 are not expressed in HEK293 (ProteinAtlas.org) cells and can therefore not affect extracellular citrate disposition in these cells.
f. HEK293 cells express *SLC25A1* at considerable levels. However, this protein is only present in mitochondria and not at the plasma membrane. The specific SLC25A1 splice variant that routes to the plasma membrane is not present in HEK293 cells. In HEK293 cells, SLC25A1 is therefore unlikely to contribute to extracellular citrate disposition[1].
g. There are no known ecto-enzymes in the extracellular milieu that metabolize citrate. Consequently, decreased metabolism cannot account for the increased levels of citrate present in medium of ANKH-containing HEK293 cells.

We now added a paragraph (**Lines 352-361)** to the discussion section detailing why increased metabolism or decreased cellular uptake cannot underlie the ANKH-dependent accumulation of nucleotides and citrate in the extracellular environment. Increased cellular release must therefore underlie the accumulation of nucleotides and TCA-cycle intermediates in the culture medium of ANKH-containing cells.

2. **“This suggests a role of citrate for bone metabolism or mirrors simply the metabolic status of the entire organism. The reviewer assumes that the measurements reflect a systemic metabolic status, which indicates an impact of ANKH on metabolic processes specifically in non-mineralized tissues.”**

We conclude that Ank has an important function in regulating local citrate disposition, including disposition in bone matrix, for the following reasons:

a. Most of the PPi present in the circulation originates from ABCC6-mediated ATP release, whereas the majority of PPi in bone tissue depends on ANKH-activity. We have preliminary data, obtained in *Abcc6^-/-^* mice, showing that Abcc6, which affects systemic PPi levels in the circulation, has only a minor effect on PPi incorporation into bone tissue. This indicates that local ANKH-activity is responsible for most of PPi found in bone tissue. As both, ATP and citrate, leave cells in an ANKH-dependent manner, also citrate levels in bone will for a large part depend on local ANKH-activity. However, citrate present in the circulation most likely also contributes to citrate incorporation into bone. A conditional Ank knockout mouse model might in the future shed more light on the relative contribution of systemic and local citrate release on citrate levels in bone. It is even possible that part of citrate levels in the circulation partly depend on citrate released during bone remodeling.
b. Plasma citrate might, however, also contribute to citrate incorporation into bone tissue. Likewise, plasma PPi most likely somewhat contributes to PPi levels in bone matrix.
c. ANKH is expressed at high levels in osteoblast, which are known to release citrate.
d. The joint space is not in direct contact with the blood circulation, but yet PPi levels depend on ANKH activity. This indicates that ANKH is an important regulator of local extracellular PPi and, possibly citrate, concentrations and that ANKH does not predominantly act via altering systemic metabolic status.

We briefly mention these points in the discussion section (**lines 315-320, 287-288**). Analysis of a conditional Ank mouse mutant might in the future elucidate the exact contribution of systemic/local citrate and PPi to the disposition of these compounds in bone matrix.

3. **The measured bone parameter in *Ank^ank/ank^* mice do not specify were the measurements have been done**

We apologize for this oversight. The methods now state: *“Cortical bone was analyzed using a 1 mm thick region of interest centered at the mid-diaphysis of the femur. Quantitative analysis was performed in accordance with the recommendations of the American Society for Bone and Mineral Research” [****PMID: 20533309]* Discussion, lines 489-492**

4. **The authors should follow the ASBMR nomenclature guidelines for bone measurements (PMID 23197339, 3455637).**

We have carefully reviewed our manuscript to ensure that our nomenclature is accurate. Importantly, **we have not performed any dynamic or static histomorphometric analyses in this paper.** As a result, the nomenclature utilized in this paper conforms to the ASBMR standard for µCT analysis (PMID: 20533309).

5. **The authors should set their long bone related findings in the context of the literature related to the *Ank^ank/ank^* bone phenotype (e.g. PMID 15833911, 20200976, 20943778). At the used age, *Ank^ank/ank^* mice have established the full ankylosing spondylitis phenotype. The authors ignore this aspect.**

In the revised version of our manuscript, we briefly discuss our long bone findings in relation to observations of other groups regarding this subject and refer to the articles suggested by reviewer 1: **Discussion,** **lines 321-325**

6. **The biological role of PPi and bisphosphonates should not mixed up!**

We apologize for the confusion caused. We are aware of the different mechanism by which PPi and bisphosphonates affect bone mineral density and aimed to mention that PPi and bisphosphonates had a similar effect on bone mineral density, i.e. both compounds increase bone mineral density. By no means we aimed to suggest both compounds have the same mechanism of action. We have changed the wording in the discussion section and mention that both compounds have a clearly different way of action. **Discussion, line 330**

7. **The discussion of citrate and ANKH in other conditions (mental retardation, sperm formation) is speculative.**

We agree with the reviewer that specific statements about potential functions of ANKH in brain and sperm are premature. In the revised version of our manuscript, we have removed these statements and substantially reduced speculation about additional functions of ANKH.

**Technical criticism**

8. **Fig1A. The shown Western blot is not very convincing. The reviewer is aware that detection of ANKH is difficult. Loading control of reference proteins is missing. Quantitative PCR would be important to verify ANKH transcript levels in the generated cell lines.**

We have used Na^+^/K^+^-ATPase immunoblot analysis and ponceau A staining of the immunoblot shown in Figure 1A, to show equal loading. These data have been added to the manuscript as supplemental data (Supplemental Figure 1). In supplemental figure 1 we also present the complete film of the immunoblot analysis for ANKH shown in Figure 1A.

9. **Fig1B-D. The shown PPi, AMP and RLU (ATP release) levels are not normalized to cell/protein content.**

We are happy to clarify this issue. For the data shown in figures 1 (panels B and C) and 2 (all panels), equal numbers of cells were seeded/well (500,000/well). In addition, at the start of each experiment, the wells were checked visually for the presence of a confluent monolayer. Finally, equal cell density of confluent monolayers was confirmed by quantifying intracellular ATP levels. Increased PPi, AMP and ATP levels in the culture medium must therefore be a consequence of ANKH activity and are not due to differences in cell density. We have extended the description of these experiments in the materials and methods section, now detailing these issues. **Supplemental figure 2 has been added to show equal intracellular ATP levels**. **Materials and Methods, lines 74-76 & 78-80.**

10. **Fig2A-C. Metabolic measurements have not been normalized to cell number or protein level. Please show values of cell line ANKHL244S B1.**

For normalization see point 9. We have analyzed several independent HEK293 cell clones for PPi concentrations in medium. These clones gave very similar results as those shown in Figure 1. As requested by the reviewer, we present extracellular PPi and citrate concentrations in medium of HEK293 cells overproducing ANKH-L244S clone B1 as **Supplemental Figure 3**.

11. **Fig3A-C. Metabolic measurements have not been normalized to cell number or protein level. Please show values of cell line ANKHL244S B1.**

For normalization see point 9. For citrate concentrations in medium of HEK293 cells overproducing ANKH-L244S clone B1 see Supplemental Figure 3B

12. **The L244S mutation in ANKH is a special mutation showing high homology with sign of *Ank^ank/ank^* mice (PMID 20943778).**

We apologize for this mistake. In the revised version of our manuscript we describe this patient as ”a human patient with biallelic inactivating mutations in ANKH”.

13. **Fig6A-F. Show clearly where the measurements have been done (site of bone). microCT should easily provide the images to demonstrate overall bone structure and used ROI.**

We have added the specific location/ROI for the measurements presented in panels A-F of figure 6. **Discussion, lines 489-492**

14. **Fig6G. The analysis reveals the overall bone strength. This type of analysis is not appropriated to differentiate the impact of composite bone components collagen, ECM, and mineral phase.**

We do not completely understand what the reviewer means. We do not attempt to ascribe a differential impact of various bone components to strength, only that overall bone strength is diminished.

**Minor criticism**

15. **The used gene/protein nomenclature should be consistently according to Ensemble and NCBI. *Ank* - mouse gene, Ank - mouse protein, *ANKH* - human gene, ANKH - human protein. This makes reading easier. ANKH/ANK is not necessary.**

We have changed nomenclature according to the suggestion of reviewer 1

16. **For the mouse model used I would use a consistent nomenclature as well. For instance, *Ank^wt/wt^, Ank^wt/ank^, Ank^ank/ank^*.**

We have changed nomenclature in the figures according to the suggestions of reviewer 1

**Reviewer 2**

**Major criticism**

1. **The conclusion that ANKH does not transport pyrophosphate.**

Our data indicate that NTP release underlies most of the PPi in the extracellular environment that depends on ANKH-activity is derived from released NTPs. We agree with reviewer 2 that our current data do not completely exclude the possibility that small amounts of pyrophosphate leave cell via ANKH. We have therefore toned down the statement that ANKH does not transport pyrophosphate. In the revised version of the manuscript we now conclude that release of NTPs underlies most of the extracellular PPi that depends on ANKH-activity. **Discussion, lines 302-304.**

Our conclusion that Ank does not transport pyrophosphate was also based on our recent unpublished analyses of *Enpp1^-/-^* mice. PPi is virtually absent in bones of these mice (<2% of the levels found in wild type mice). The work presented in our current study shows that 75% of the PPi in bone depends on Ank-activity. This means that the PPi that ends up in bone via Ank requires Enpp1 activity. Consequently, NTP release must underly virtually all PPi present in bones of wild type mice. Unfortunately, due to the current situation regarding covid-19 we can at present not follow up on these observations in the *Enpp1^-/-^* mice. We hope to do so in the future.

We also agree with reviewer 2 that the data describing ANKH-dependent transport of PPi comes from a reputed laboratory. It is important to note, however, that the authors were unable to demonstrate ANKH-dependent efflux of PPi in their frog oocyte experiments [2]. Instead, they followed ANKH-dependent uptake in frog oocytes. It is technically challenging to reliably quantify intracellular PPi concentrations, because of the abundant presence of cytosolic pyrophosphatases (PPA1 and PPA2 [3]). These technical challenges also prevent us to reliably determine intracellular pyrophosphate concentrations in HEK293 cells, as proposed by reviewer 2.

Of note, many biochemical reactions, including those involved in the synthesis of macromolecules like proteins and nucleic acids, result in the liberation of pyrophosphate. Without cytosolic pyrophosphatases, reactions underlying synthesis of macromolecules would be reversible. Intracellular concentrations of PPi need to be kept low by cytosolic pyrophosphates. There is currently no consensus as to how low cytosolic PPi are [3], but they might very well be too low to allow substantial amounts to be released into the extracellular environment.

2. **The discordance between PPi levels and AMP levels in the medium of HEK293 cells expressing ANKH is of significance.**

This is a significant point and we are happy to clarify this issue. In theory, direct transport of PPi by ANKH could also explain this result. For reasons detailed under point 1, we conclude however, that ANKH does not transport significant amounts of PPi. We explain the discordance between PPi and AMP levels in the culture medium as follows:

a) Not only extracellular ATP underlies pyrophosphate concentrations in the culture medium. Other nucleoside triphosphates, like GTP, UTP etc. also contribute to extracellular pyrophosphate.
b) Extracellular metabolism of AMP to adenosine by NT5E and will reduce extracellular AMP. We have seen in HEK293 display substantial NT5E activity as we have found in previous work [4]. Adenosine in turn can be further metabolized to inosine. Compared to AMP, extracellular PPi is more stable, as HEK293 cells virtually lack TNAP, the major enzyme involved in pyrophosphate degradation (our unpublished observations).

We have extended our explanation for the lower AMP than pyrophosphate concentrations in the results section and now refer to previous work showing considerable metabolism of extracellular AMP by HEK293 cells. **Materials and Methods, lines 480-482**

3. **The authors attempt to explain the difference in citrate in mouse urine and in urine with biallelic mutations in ANKH with potential differences in dietary citrate. This conclusion seems arbitrary.**

We agree with reviewer 2 that differences in dietary citrate is only one of several potential explanations. We do not intend to suggest this is the only possible explanation and have slightly changed the wording of this statement. Moreover, citrate not necessarily needs to be completely absent in plasma of the patient with biallelic mutations in ANKH. The acid-base status in plasma is, for instance, also an important factor in urinary citrate excretion. No plasma sample of this patient is available to us and we are therefore unable to determine citrate plasma levels in patients with mutations in ANKH. We added a few other explanations to explain the difference in citrate levels between mice and humans. **Discussion, lines 277-285**.

4. **What could be the role of ANKH in renal handling of citrate and hence in the presence or absence of citrate in urine?**

This is an interesting question. Next to citrate plasma levels, excretion into urine is highly dependent on the pH of primary urine and hence the acid-base status: SLC13A5 is a crucial factor in the uptake of citrate from primary urine. SLC13A5 transports the divalent form of citrate. Uptake of citrate from primary urine therefore depends on pH, with less citrate being reabsorbed at higher pH. Vice versa, at low pH more citrate will be reabsorbed and less will end up in urine. Most of the citrate that is reabsorbed will be metabolized intracellularly. For these reasons, we expect that Ank affects citrate excretion mostly by regulating citrate plasma concentrations and not by directly mediating release of citrate from the kidney. There might be a small contribution of ANKH-mediated release directly from the cells of the proximal tubule to urinary citrate excretion. We have adapted the text in the discussion section accordingly. **Discussion, 282-285**.

Minor points

5. **Page 4: ENPP1 is written as "ecto-nucleotidase pyrophosphatase/ phosphodiesterase 1" It is "ecto-nucleotide pyrophosphatase/ phosphodiesterase.**

We apologize for this mistake. Corrected as suggested by reviewer 2

6. **Page 4, second para, line 3: change "which" to "with".**

Corrected as suggested by reviewer 2

7. **Page 11: citrate is not found at 180 mM in sperm. This concentration should refer to seminal plasma, not sperm.**

Thank you for noting this mistake. We have removed the extensive discussion of a potential function of Ank/extracellular citrate in sperm physiology, as requested by reviewer 1.

**Reviewer 3**

1. **Figure 1 Done in 293 cells but well controlled. The data look convincing but could the authors include an indication of the P values in the Figure or Figure legend at least for the final time point?**

According to the suggestions of the reviewer, we have calculated statistical significance for the data presented in panels B, C and D of Figure 1. **Statistical significance is now indicated in panels B, C and D of Figure 1.**

2. **Figure 2 AMP highly significant. Is ENPP1 activity high though in relevant tissues such as bone and muscle for example, if so, this would add weight to the authors’ hypothesis that PPi comes from the conversion of NTPs to NMPs**

ENPP1 is widely expressed, and especially abundant in bone tissue, which we now mention in the discussion section. **Discussion, lines 313-314**.

3. **Could the authors give more details of the culture methods used and in particular whether the DMEM High clone medium and the antibiotics contained citrate?**

We were unaware of the fact that many penicillin/streptomycin formulations contain citrate. Our efflux experiments were performed in serum-free Pro293a (Lonza) medium supplemented with L-glutamine and penicillin/streptomycin (Gibco). According to the manufacturer (Gibco), this antibiotic formulation does not contain citrate, sodium chloride. We have added the manufacturer of the pen/strep solution to the materials and methods section as it is essential to use citrate-free antibiotics to the culture medium **Materials and Methods, lines 377**. Absence of citrate in penicillin and streptomycin containing Pro293a medium was also apparent from LC/MS-based and enzymatic citrate assays of medium used in the efflux experiments.

4. **The importance of citrate in disease is largely understated in the discussion.**

We have put more emphasis on the important functions of citrate in disease, without being too speculative. Moreover, we now cite the suggested papers. **Discussion, lines 261-269**.

5. **There is no previous report of a mammalian malate transporter and this should be mentioned.**

We thank reviewer 3 for bringing to our attention that no mammalian efflux transporter for malate had been described. We now clearly state in the results section we are the first to identify a plasma membrane protein being involved in the cellular release of malate (**lines 176-178**)**.** We also discuss the possibility that ANKH contributes to plasma malate. **Discussion lines 252-255**.

6. **Can the authors distinguish between citrate and isocitrate?**

Our LC/MS method does not distinguish between citrate and isocitrate. However, the enzymatic citrate assay is highly specific. We can therefore confidently conclude that in the HEK293 cells ANKH is involved in the release of citrate and not isocitrate. We now mention in the results section that the enzymatic assay is specific for citrate, confirming that ANKH-containing HEK293 cells release citrate and not isocitrate. **Results, lines 182-184**.

7. **I think citrate in the plasma is normally much lower than 300 µM.**

We have added the reference suggested by reviewer 3 and rephrased our statement about plasma citrate concentrations accordingly. **Discussion, lines 258-259**.

8. **The mechanism of citrate release by astrocytes is not known (Mycielska and Geissler Current Molecular Medicine 2015) and so do astrocytes express ANKH?**

Although ANKH is known to be expressed at relatively high levels in brain, the specific cell type expressing ANKH is unknown. As reviewer 1 found several of our statements about ANKH and citrate speculative, we have removed details about potential functions of ANKH/citrate in brain from the discussion section.

**References**

1.	Mazurek MP, Prasad PD, Gopal E, Fraser SP, Bolt L, Rizaner N, et al. Molecular origin of plasma membrane citrate transporter in human prostate epithelial cells. EMBO reports. EMBO Press; 2010;11: 431–437. doi:10.1038/embor.2010.51

2.	Gurley KA, Reimer RJ, Kingsley DM. Biochemical and genetic analysis of ANK in arthritis and bone disease. Am J Hum Genet. 2006;79: 1017–1029. doi:10.1086/509881

3.	Heinonen JK. Biological Role of Inorganic Pyrophosphate. Springer Science & Business Media; 2000.

4.	Jansen RS, Küçükosmanoglu A, de Haas M, Sapthu S, Otero JA, Hegman IEM, et al. ABCC6 prevents ectopic mineralization seen in pseudoxanthoma elasticum by inducing cellular nucleotide release. Proc Natl Acad Sci USA. 2013;110: 20206–20211. doi:10.1073/pnas.1319582110
