# Supplementary material for: The membrane protein ANKH is crucial for bone mechanical performance by mediating cellular export of citrate and ATP
Source: PLoS Genet. 2020 Jul 8;16(7):e1008884. doi: 10.1371/journal.pgen.1008884 (PMC7371198; doi:10.1371/journal.pgen.1008884)
Supplement: Peer review history

# Peer review: Decision Letter 0

**DOI:** 10.1371/journal.pgen.1008884.r001

**Authors:** Gregory S. Barsh, Maria Mycielska

**Transfer Alert**

This paper was transferred from another journal. As a result, its full editorial history (including decision letters, peer reviews and author responses) may not be present.

27 Mar 2020

Dear Dr.  Van de Wetering

Thank you very much for submitting your Research Article entitled 'The membrane protein ANKH is critical for normal development and plays a key role in the cellular export of citrate and ATP from mammalian cells' to PLOS Genetics. Your manuscript was fully evaluated at the editorial level and by independent peer reviewers. The reviewers appreciated the attention to an important problem, but raised some substantial concerns about the current manuscript. Based on the reviews, we will not be able to accept this version of the manuscript, but we would be willing to review again a much-revised version. We cannot, of course, promise publication at that time.

Should you decide to revise the manuscript for further consideration here, your revisions should address the specific points made by each reviewer. We will also require a detailed list of your responses to the review comments and a description of the changes you have made in the manuscript.

If you decide to revise the manuscript for further consideration at PLOS Genetics, please aim to resubmit within the next 60 days, unless it will take extra time to address the concerns of the reviewers, in which case we would appreciate an expected resubmission date by email to plosgenetics@plos.org.

If present, accompanying reviewer attachments are included with this email; please notify the journal office if any appear to be missing. They will also be available for download from the link below. You can use this link to log into the system when you are ready to submit a revised version, having first consulted our Submission Checklist.

To enhance the reproducibility of your results, we recommend that you deposit your laboratory protocols in protocols.io, where a protocol can be assigned its own identifier (DOI) such that it can be cited independently in the future. For instructions see our guidelines.

Please be aware that our data availability policy requires that all numerical data underlying graphs or summary statistics are included with the submission, and you will need to provide this upon resubmission if not already present. In addition, we do not permit the inclusion of phrases such as "data not shown" or "unpublished results" in manuscripts. All points should be backed up by data provided with the submission.

While revising your submission, please upload your figure files to the Preflight Analysis and Conversion Engine (PACE) digital diagnostic tool.  PACE helps ensure that figures meet PLOS requirements. To use PACE, you must first register as a user. Then, login and navigate to the UPLOAD tab, where you will find detailed instructions on how to use the tool. If you encounter any issues or have any questions when using PACE, please email us at figures@plos.org.

PLOS has incorporated Similarity Check, powered by iThenticate, into its journal-wide submission system in order to screen submitted content for originality before publication. Each PLOS journal undertakes screening on a proportion of submitted articles. You will be contacted if needed following the screening process.

To resubmit, use the link below and 'Revise Submission' in the 'Submissions Needing Revision' folder.

[LINK]

We are sorry that we cannot be more positive about your manuscript at this stage. Please do not hesitate to contact us if you have any concerns or questions.

Yours sincerely,

Maria Mycielska, PhD

Guest Editor

PLOS Genetics

Gregory Barsh

Editor-in-Chief

PLOS Genetics

Reviewer's Responses to Questions

**Comments to the Authors:**

**Please note here if the review is uploaded as an attachment.**

Reviewer #1: please see reviewer comments as attachment

Reviewer #2: This manuscript reports on some novel functions of the transmembrane protein ANKH/Ank. The most important findings include the transport of nucleoside triphosphate and the TCA cycle intermediates by ANKH/Ank.

Critique

1. A major concern is the authors' conclusion that ANKH/Ank does not transport pyrophosphate. The authors quote the original published report in the journal Science as the prior evidence for pyrophosphate transport via ANKH by other investigators. But subsequent studies seem to have established quite convincingly that ANKH does transport pyrophosphate using heterologous expression in canopus leaves oocytes. As this is one of the major issues focused in this manuscript, it would be nice of the authors could use their HEK293 cells over expressing wild type ANKH to monitor pyrophosphate uptake. Previous studies from other laboratories have shown that frog oocytes expressing ANKH mediate pyrophosphate uptake; the data from these studies seem convincing. It is possible that the conclusions reported in the current manuscript are correct, but this needs further verification.

2. With reference to the concern raised above, the discordance between PPi levels and AMP levels in the medium of HEK293 cells expressing ANKH is of signficance. PPi levels are twice that of AMP levels. Even when the levels of other NMPs are taken into account, still PPi levels are significantly higher than the cumulative levels of all NMPs combined. This discrepancy needs explanation. What if ANKH transports not only NTPs but also PPi? Would this explain the discrepancy?

3. Fig. 5. Urine from patients with ANKH mutation has no citrate whereas urine from and/ank mutant mice show significant levels of citrate. The authors attempt to explain this difference with potential differences in dietary citrate. This conclusion seems arbitrary. This would mean that the plasma of this particular patient had no citrate at all, thus explaining the absence of citrate in urine. is this true? Of course, the ankh/ank mice do show citrate in plasma and therefore, the authors could argue that urine shows citrate. But this will depend upon whether or not the same explanation is true for plasma from the human patient. For the authors' argument to be correct, the patient plasma should not have citrate. This seems very unlikely.

4. Other studies have shown that ANKH is expressed in kidney and in kidney epithelial cells. In fact, the protein is expressed in the plasma membrane of these cells. What could be the role of ANKH in renal handling of citrate and hence in the presence or absence of citrate in urine?

Minor comments

1. Page 4: ENPP1 is written as "ecto-nucleotidase pyrophosphatase/phosphodiesterase 1" It is "ecto-nucleotide pyrophosphatase/phosphodiesterase 1. This term has been used correctly in Authors Summary.

2. Page 4, second para, line 3: change "which" to "with".

3. Page 11: citrate is not found at 180 mM in sperm. This concentration should refer to seminal plasma, not sperm.

Reviewer #3: Szeri et al present some very interesting findings on the release of inorganic phosphate, citrate and to a lesser extent malate and succinate via membrane transporter protein ANKH. This reviewer is not an expert on bone function but the findings will be of great interest to investigators in many fields as plasma citrate has been implicated in ageing, type 2 diabetes, neuronal function and cancer but knowledge of its efflux from cells is incomplete. Similarly, as far as I am are the investigators are the first to provide evidence for a transporter of malate efflux in mammalian cells and plasma malate has been implicated in ageing and cardiovascular disease.

In general, the studies have been well conducted, the data look straightforward and convincing.

Results

Figure 1 Done in 293 cells but well controlled. The data look convincing but could the authors include an indication of the P values in the Figure or Figure legend at least for the final time point?

Figure 2 AMP highly significant. Is ENPP1 activity high though in relevant tissues such as bone and muscle for example, if so, this would add weight to the authors’ hypothesis that PPi comes from the conversion of NTPs to NMPs.

Figure 5 Line 207 the reference to succinate should I think read Figure 5C and what about malate?

Methodology

Could the authors give more details of the culture methods used and in particular whether the DMEM High clone medium and the antibiotics contained citrate? This is important because some suppliers provide antibiotics in citrate buffer.

LC/MS and NMR Can the authors distinguish between citrate and isocitrate with the methods they have used? If not this should be made clear in the methods section and in the discussion.

Discussion

This could be improved as it largely understates the importance of citrate in disease.

The citrate importer SLC13A5 knockout mouse is resistant to type 2 diabetes (Birkenfeld et al Cell Metabolism 2011) and essential for normal neuronal functioning in humans (Mycielska and Geissler Current Molecular Medicine 2015). Furthermore, citrate via pmCiC has been implicated in cancer growth (Mycielska et al 2018) and so these papers also add weight to the authors suggestion that extracellular citrate and ANKH could have a more widespread role in human pathologies and could be cited.

ANKH appears to mediate the secretion of malate and succinate as well as citrate. Although the levels of malate and succinate secretion are less then citrate, as far as I am aware there is no previous report of a mammalian malate transporter and this should be mentioned as it could explain the widespread reports of malate in human plasma.

Can the authors distinguish between citrate and isocitrate? These issues are important because citrate/isocitrate and phosphate have been reported to be two of the 22 metabolites linked to chronological age independently of BMI, blood pressure, fev, liver function and telomere length (Menni et al Int. J. Epidemiol. 2013) and isocitrate and malate are inversely associated with longevity and are associated with increased cardiovascular disease risk (Cheng et al Nature Communications 2015). Isocitrate is more significant than malate. These studies also support the authors speculation that ANKH might have widespread biological effects beyond bone.

Line 249 and reference 19 I think citrate in the plasma is normally much lower than 300µM in healthy people the range quoted in another review was 52-106 µM but it doubles in diseases like non-alcoholic fatty acid disease (Iacobazzi and Infantino Biol. Chem. 2014).

Line 269 The mechanism of citrate release by astrocytes is not known (Mycielska and Geissler Current Molecular Medicine 2015) and so do astrocytes express ANKH?

**********

**Have all data underlying the figures and results presented in the manuscript been provided?**

Large-scale datasets should be made available via a public repository as described in the *PLOS Genetics* data availability policy, and numerical data that underlies graphs or summary statistics should be provided in spreadsheet form as supporting information.

Reviewer #1: Yes

Reviewer #2: Yes

Reviewer #3: Yes

**********

PLOS authors have the option to publish the peer review history of their article (what does this mean?). If published, this will include your full peer review and any attached files.

If you choose “no”, your identity will remain anonymous but your review may still be made public.

**Do you want your identity to be public for this peer review?** For information about this choice, including consent withdrawal, please see our Privacy Policy.

Reviewer #1: Yes: Jirko Kühnisch

Reviewer #2: No

Reviewer #3: No

Attachment Submitted filename: 200319_Review_Plos_Genetics_ANKH_citrate.docx Click here for additional data file.

# Author comment: Author response to Decision Letter 0

**DOI:** 10.1371/journal.pgen.1008884.r002

29 Apr 2020

Attachment Submitted filename: Response to reviewers.docx Click here for additional data file.

# Peer review: Decision Letter 1

**DOI:** 10.1371/journal.pgen.1008884.r003

**Authors:** Gregory S. Barsh, Maria Mycielska

25 May 2020

Dear Dr. Van de Wetering,

We are pleased to inform you that your manuscript entitled "The membrane protein ANKH is crucial for bone mechanical performance by mediating  cellular export of citrate and ATP" has been editorially accepted for publication in PLOS Genetics. Congratulations!

Before your submission can be formally accepted and sent to production you will need to complete our formatting changes, which you will receive in a follow up email. Please be aware that it may take several days for you to receive this email; during this time no action is required by you. Please note: the accept date on your published article will reflect the date of this provisional accept, but your manuscript will not be scheduled for publication until the required changes have been made.

Once your paper is formally accepted, an uncorrected proof of your manuscript will be published online ahead of the final version, unless you’ve already opted out via the online submission form. If, for any reason, you do not want an earlier version of your manuscript published online or are unsure if you have already indicated as such, please let the journal staff know immediately at plosgenetics@plos.org.

In the meantime, please log into Editorial Manager at https://www.editorialmanager.com/pgenetics/, click the "Update My Information" link at the top of the page, and update your user information to ensure an efficient production and billing process. Note that PLOS requires an ORCID iD for all corresponding authors. Therefore, please ensure that you have an ORCID iD and that it is validated in Editorial Manager. To do this, go to ‘Update my Information’ (in the upper left-hand corner of the main menu), and click on the Fetch/Validate link next to the ORCID field.  This will take you to the ORCID site and allow you to create a new iD or authenticate a pre-existing iD in Editorial Manager.

If you have a press-related query, or would like to know about one way to make your underlying data available (as you will be aware, this is required for publication), please see the end of this email. If your institution or institutions have a press office, please notify them about your upcoming article at this point, to enable them to help maximise its impact. Inform journal staff as soon as possible if you are preparing a press release for your article and need a publication date.

Thank you again for supporting open-access publishing; we are looking forward to publishing your work in PLOS Genetics!

Yours sincerely,

Maria Mycielska, PhD

Guest Editor

PLOS Genetics

Gregory Barsh

Editor-in-Chief

PLOS Genetics

www.plosgenetics.org

Twitter: @PLOSGenetics

----------------------------------------------------

Comments from the reviewers (if applicable):

Reviewer's Responses to Questions

**Comments to the Authors:**

**Please note here if the review is uploaded as an attachment.**

Reviewer #1: Summary

The presented revised manuscript describes metabolic abnormalities of malate/citrate and nucleotide metabolism. These findings are set into the context of Ankank/ank mice bone biology.

The reviewer is still highly positive for the novel finding of altered citrate levels after deactivation of ANKH in vitro and in vivo. This study could potentially provide first evidence linking general metabolism with ANKH associated phenotypes

The criticism of the reviewer was addressed by the authors. The authors add 3 supplementary figures. However, none of the presented data (figures) were significantly adapted or expanded to improve presentation, information and scientific basis of conclusion.

Major Criticism

The authors adapted the title of the manuscript. Now, the authors link altered citrate and ATP export to altered bone mechanical performance. This title is not justified by the presented mechanical and structural bone data. The structural bone data focus on cortical bone and are incomplete. The mechanical bone data are insufficient to identify differences between the collagenous, ECM and mineral phase. The accumulated bone and mechanical bone data cannot be linked to altered citrate levels. Moreover, the title suggests the cellular export of citrate and ATP directly impacts bone mechanics. For such a statement bone cells e.g. osteoblasts need to be analyzed.

The main source of systemic citrate are metabolic highly active tissues such as liver or skeletal muscle. Thus, the cause of altered systemic citrate levels was not clarified by the study. Mixing up Hek293 in vitro and incomplete bone data is not sufficiently answering the topic of the study. The study is not able to differentiate between local and systemic effects of altered metabolism

The authors statement “Our data indicate reduced levels of extracellular citrate at least partly underly the observed delay in osteogenic differentiation of Ankank/ank osteoblast.” is wrong. They did not analyze osteoblasts.

The authors statement “Possibly, also part of the ankylosis inhibitory effect of ANKH might come from citrate released into the joint space.” is highly speculative.

The discussion of the bone findings is superficial and not adequate to the manuscript title. The discussion of citrate in bone is not adequate to the literature. Role of citrate and bone (PMID 31731473).

Technical Criticism

Fig1A. Loading control of reference proteins were added. The authors did not improve quality of ANKH detection. Quantitative PCR to verify ANKH transcript levels in the generated cell lines was not added.

The authors comment on the cell approach e.g. by seeding 500 K cells/well. The authors did not normalize their generated data to the final cell number/protein content/ DNA amount/ ATP content. Presenting cell experimental without normalization is not state of the art.

The authors provide a statement where the measurement has been done. The authors did not improve the figure by adding images of the measured bone site and the observed phenotype. All these data are easily available and can be published.

The authors did not show the complete list of bone measurement parameters. This is not state of the art and makes the data not fully interpretable. The graphs do not use the standard abbreviation for microCT bone parameter. The material and methods do not provide the calibration approach for bone density measurement. What means the parameter eccentricity? Which bone perimeter value is used Ps.Pm or Ec.Pm?

The authors did not expand the data for bone mechanical analysis, which may help to differentiate effects of the ECM, collagenous matrix or mineral phase. These data are essential to identify the impact of altered PPi and citrate levels.

Reviewer #2: The authors have addressed all of the comments raised during the initial review.

Reviewer #3: The authors have addressed all of my questions satisfactorily.

**********

**Have all data underlying the figures and results presented in the manuscript been provided?**

Large-scale datasets should be made available via a public repository as described in the *PLOS Genetics* data availability policy, and numerical data that underlies graphs or summary statistics should be provided in spreadsheet form as supporting information.

Reviewer #1: Yes

Reviewer #2: Yes

Reviewer #3: Yes

**********

PLOS authors have the option to publish the peer review history of their article (what does this mean?). If published, this will include your full peer review and any attached files.

If you choose “no”, your identity will remain anonymous but your review may still be made public.

**Do you want your identity to be public for this peer review?** For information about this choice, including consent withdrawal, please see our Privacy Policy.

Reviewer #1: Yes: Jirko Kühnisch

Reviewer #2: Yes: Vadivel Ganapathy

Reviewer #3: No

----------------------------------------------------

**Data Deposition**

If you have submitted a Research Article or Front Matter that has associated data that are not suitable for deposition in a subject-specific public repository (such as GenBank or ArrayExpress), one way to make that data available is to deposit it in the Dryad Digital Repository. As you may recall, we ask all authors to agree to make data available; this is one way to achieve that. A full list of recommended repositories can be found on our website.

The following link will take you to the Dryad record for your article, so you won't have to re‐enter its bibliographic information, and can upload your files directly: 

http://datadryad.org/submit?journalID=pgenetics&manu=PGENETICS-D-20-00369R1

More information about depositing data in Dryad is available at http://www.datadryad.org/depositing. If you experience any difficulties in submitting your data, please contact help@datadryad.org for support.

Additionally, please be aware that our data availability policy requires that all numerical data underlying display items are included with the submission, and you will need to provide this before we can formally accept your manuscript, if not already present.

----------------------------------------------------

**Press Queries**

If you or your institution will be preparing press materials for this manuscript, or if you need to know your paper's publication date for media purposes, please inform the journal staff as soon as possible so that your submission can be scheduled accordingly. Your manuscript will remain under a strict press embargo until the publication date and time. This means an early version of your manuscript will not be published ahead of your final version. PLOS Genetics may also choose to issue a press release for your article. If there's anything the journal should know or you'd like more information, please get in touch via plosgenetics@plos.org.

# Editor report: Acceptance letter

**DOI:** 10.1371/journal.pgen.1008884.r004

**Authors:** Gregory S. Barsh, Maria Mycielska

1 Jul 2020

PGENETICS-D-20-00369R1 

The membrane protein ANKH is crucial for bone mechanical performance by mediating  cellular export of citrate and ATP 

Dear Dr van de Wetering, 

We are pleased to inform you that your manuscript entitled "The membrane protein ANKH is crucial for bone mechanical performance by mediating  cellular export of citrate and ATP" has been formally accepted for publication in PLOS Genetics! Your manuscript is now with our production department and you will be notified of the publication date in due course.

The corresponding author will soon be receiving a typeset proof for review, to ensure errors have not been introduced during production. Please review the PDF proof of your manuscript carefully, as this is the last chance to correct any errors. Please note that major changes, or those which affect the scientific understanding of the work, will likely cause delays to the publication date of your manuscript. 

Soon after your final files are uploaded, unless you have opted out or your manuscript is a front-matter piece, the early version of your manuscript will be published online. The date of the early version will be your article's publication date. The final article will be published to the same URL, and all versions of the paper will be accessible to readers.

Thank you again for supporting PLOS Genetics and open-access publishing. We are looking forward to publishing your work! 

With kind regards,

Kaitlin Butler

PLOS Genetics

On behalf of:

The PLOS Genetics Team

Carlyle House, Carlyle Road, Cambridge CB4 3DN | United Kingdom

plosgenetics@plos.org | +44 (0) 1223-442823

plosgenetics.org | Twitter: @PLOSGenetics
